# Search for standard model production of four top quarks with same-sign and multilepton final states in proton–proton collisions at $\sqrt{s} = 13\,\text {TeV}$

**DOI:** 10.1140/epjc/s10052-018-5607-5

**Published:** 2018-02-19

**Authors:** A. M. Sirunyan, A. Tumasyan, W. Adam, F Ambrogi, E. Asilar, T. Bergauer, J. Brandstetter, E. Brondolin, M. Dragicevic, J. Erö, A. Escalante Del Valle, M. Flechl, M. Friedl, R. Frühwirth, V. M. Ghete, J. Grossmann, J. Hrubec, M. Jeitler, A. König, N. Krammer, I. Krätschmer, D. Liko, T. Madlener, I. Mikulec, E. Pree, N. Rad, H. Rohringer, J. Schieck, R. Schöfbeck, M. Spanring, D. Spitzbart, A. Taurok, W. Waltenberger, J. Wittmann, C.-E. Wulz, M. Zarucki, V. Chekhovsky, V. Mossolov, J. Suarez Gonzalez, E. A. De Wolf, D. Di Croce, X. Janssen, J. Lauwers, M. Van De Klundert, H. Van Haevermaet, P. Van Mechelen, N. Van Remortel, S. Abu Zeid, F. Blekman, J. D’Hondt, I. De Bruyn, J. De Clercq, K. Deroover, G. Flouris, D. Lontkovskyi, S. Lowette, I. Marchesini, S. Moortgat, L. Moreels, Q. Python, K. Skovpen, S. Tavernier, W. Van Doninck, P. Van Mulders, I. Van Parijs, D. Beghin, B Bilin, H. Brun, B. Clerbaux, G. De Lentdecker, H. Delannoy, B. Dorney, G. Fasanella, L. Favart, R. Goldouzian, A. Grebenyuk, A. K. Kalsi, T. Lenzi, J. Luetic, T. Maerschalk, A. Marinov, T. Seva, E. Starling, C. Vander Velde, P. Vanlaer, D. Vannerom, R. Yonamine, F. Zenoni, T. Cornelis, D. Dobur, A. Fagot, M. Gul, I. Khvastunov, D. Poyraz, C. Roskas, S. Salva, D. Trocino, M. Tytgat, W. Verbeke, N. Zaganidis, H. Bakhshiansohi, O. Bondu, S. Brochet, G. Bruno, C. Caputo, A. Caudron, P. David, S. De Visscher, C. Delaere, M. Delcourt, B. Francois, A. Giammanco, M. Komm, G. Krintiras, V. Lemaitre, A. Magitteri, A. Mertens, M. Musich, K. Piotrzkowski, L. Quertenmont, A. Saggio, M. Vidal Marono, S. Wertz, J. Zobec, W. L. Aldá Júnior, F. L. Alves, G. A. Alves, L. Brito, G. Correia Silva, C. Hensel, A. Moraes, M. E. Pol, P. Rebello Teles, E. Belchior Batista Das Chagas, W. Carvalho, J. Chinellato, E. Coelho, E. M. Da Costa, G. G. Da Silveira, D. De Jesus Damiao, S. Fonseca De Souza, L. M. Huertas Guativa, H. Malbouisson, M. Melo De Almeida, C. Mora Herrera, L. Mundim, H. Nogima, L. J. Sanchez Rosas, A. Santoro, A. Sznajder, M. Thiel, E. J. Tonelli Manganote, F. Torres Da Silva De Araujo, A. Vilela Pereira, S. Ahuja, C. A. Bernardes, T. R. Fernandez Perez Tomei, E. M. Gregores, P. G. Mercadante, S. F. Novaes, Sandra S. Padula, D. Romero Abad, J. C. Ruiz Vargas, A. Aleksandrov, R. Hadjiiska, P. Iaydjiev, M. Misheva, M. Rodozov, M. Shopova, G. Sultanov, A. Dimitrov, L. Litov, B. Pavlov, P. Petkov, W. Fang, X. Gao, L. Yuan, M. Ahmad, J. G. Bian, G. M. Chen, H. S. Chen, M. Chen, Y. Chen, C. H. Jiang, D. Leggat, H. Liao, Z. Liu, F. Romeo, S. M. Shaheen, A. Spiezia, J. Tao, C. Wang, Z. Wang, E. Yazgan, T. Yu, H. Zhang, J. Zhao, Y. Ban, G. Chen, J. Li, Q. Li, S. Liu, Y. Mao, S. J. Qian, D. Wang, Z. Xu, F. Zhang, Y. Wang, C. Avila, A. Cabrera, L. F. Chaparro Sierra, C. Florez, C. F. González Hernández, J. D. Ruiz Alvarez, M. A. Segura Delgado, B. Courbon, N. Godinovic, D. Lelas, I. Puljak, P. M. Ribeiro Cipriano, T. Sculac, Z. Antunovic, M. Kovac, V. Brigljevic, D. Ferencek, K. Kadija, B. Mesic, A. Starodumov, T. Susa, M. W. Ather, A. Attikis, G. Mavromanolakis, J. Mousa, C. Nicolaou, F. Ptochos, P. A. Razis, H. Rykaczewski, M. Finger, M. Finger, E. Carrera Jarrin, Y. Assran, S. Elgammal, A. Mahrous, S. Bhowmik, R. K. Dewanjee, M. Kadastik, L. Perrini, M. Raidal, A. Tiko, C. Veelken, P. Eerola, H. Kirschenmann, J. Pekkanen, M. Voutilainen, J. Havukainen, J. K. Heikkilä, T. Järvinen, V. Karimäki, R. Kinnunen, T. Lampén, K. Lassila-Perini, S. Laurila, S. Lehti, T. Lindén, P. Luukka, T. Mäenpää, H. Siikonen, E. Tuominen, J. Tuominiemi, T. Tuuva, M. Besancon, F. Couderc, M. Dejardin, D. Denegri, J. L. Faure, F. Ferri, S. Ganjour, S. Ghosh, A. Givernaud, P. Gras, G. Hamel de Monchenault, P. Jarry, I. Kucher, C. Leloup, E. Locci, M. Machet, J. Malcles, G. Negro, J. Rander, A. Rosowsky, M. Ö. Sahin, M. Titov, A. Abdulsalam, C. Amendola, I. Antropov, S. Baffioni, F. Beaudette, P. Busson, L. Cadamuro, C. Charlot, R. Granier de Cassagnac, M. Jo, S. Lisniak, A. Lobanov, J. Martin Blanco, M. Nguyen, C. Ochando, G. Ortona, P. Paganini, P. Pigard, R. Salerno, J. B. Sauvan, Y. Sirois, A. G. Stahl Leiton, T. Strebler, Y. Yilmaz, A. Zabi, A. Zghiche, J.-L. Agram, J. Andrea, D. Bloch, J.-M. Brom, M. Buttignol, E. C. Chabert, N. Chanon, C. Collard, E. Conte, X. Coubez, F. Drouhin, J.-C. Fontaine, D. Gelé, U. Goerlach, M. Jansová, P. Juillot, A.-C. Le Bihan, N. Tonon, P. Van Hove, S. Gadrat, S. Beauceron, C. Bernet, G. Boudoul, R. Chierici, D. Contardo, P. Depasse, H. El Mamouni, J. Fay, L. Finco, S. Gascon, M. Gouzevitch, G. Grenier, B. Ille, F. Lagarde, I. B. Laktineh, M. Lethuillier, L. Mirabito, A. L. Pequegnot, S. Perries, A. Popov, V. Sordini, M. Vander Donckt, S. Viret, S. Zhang, A. Khvedelidze, I. Bagaturia, C. Autermann, L. Feld, M. K. Kiesel, K. Klein, M. Lipinski, M. Preuten, C. Schomakers, J. Schulz, M. Teroerde, B. Wittmer, V. Zhukov, A. Albert, D. Duchardt, M. Endres, M. Erdmann, S. Erdweg, T. Esch, R. Fischer, A. Güth, T. Hebbeker, C. Heidemann, K. Hoepfner, S. Knutzen, M. Merschmeyer, A. Meyer, P. Millet, S. Mukherjee, T. Pook, M. Radziej, H. Reithler, M. Rieger, F. Scheuch, D. Teyssier, S. Thüer, G. Flügge, B. Kargoll, T. Kress, A. Künsken, T. Müller, A. Nehrkorn, A. Nowack, C. Pistone, O. Pooth, A. Stahl, M. Aldaya Martin, T. Arndt, C. Asawatangtrakuldee, K. Beernaert, O. Behnke, U. Behrens, A. Bermúdez Martínez, A. A. Bin Anuar, K. Borras, V. Botta, A. Campbell, P. Connor, C. Contreras-Campana, F. Costanza, C. Diez Pardos, G. Eckerlin, D. Eckstein, T. Eichhorn, E. Eren, E. Gallo, J. Garay Garcia, A. Geiser, J. M. Grados Luyando, A. Grohsjean, P. Gunnellini, M. Guthoff, A. Harb, J. Hauk, M. Hempel, H. Jung, M. Kasemann, J. Keaveney, C. Kleinwort, I. Korol, D. Krücker, W. Lange, A. Lelek, T. Lenz, J. Leonard, K. Lipka, W. Lohmann, R. Mankel, I.-A. Melzer-Pellmann, A. B. Meyer, M. Missiroli, G. Mittag, J. Mnich, A. Mussgiller, E. Ntomari, D. Pitzl, A. Raspereza, M. Savitskyi, P. Saxena, R. Shevchenko, N. Stefaniuk, G. P. Van Onsem, R. Walsh, Y. Wen, K. Wichmann, C. Wissing, O. Zenaiev, R. Aggleton, S. Bein, V. Blobel, M. Centis Vignali, T. Dreyer, E. Garutti, D. Gonzalez, J. Haller, A. Hinzmann, M. Hoffmann, A. Karavdina, R. Klanner, R. Kogler, N. Kovalchuk, S. Kurz, T. Lapsien, D. Marconi, M. Meyer, M. Niedziela, D. Nowatschin, F. Pantaleo, T. Peiffer, A. Perieanu, C. Scharf, P. Schleper, A. Schmidt, S. Schumann, J. Schwandt, J. Sonneveld, H. Stadie, G. Steinbrück, F. M. Stober, M. Stöver, H. Tholen, D. Troendle, E. Usai, A. Vanhoefer, B. Vormwald, M. Akbiyik, C. Barth, M. Baselga, S. Baur, E. Butz, R. Caspart, T. Chwalek, F. Colombo, W. De Boer, A. Dierlamm, N. Faltermann, B. Freund, R. Friese, M. Giffels, M. A. Harrendorf, F. Hartmann, S. M. Heindl, U. Husemann, F. Kassel, S. Kudella, H. Mildner, M. U. Mozer, Th. Müller, M. Plagge, G. Quast, K. Rabbertz, M. Schröder, I. Shvetsov, G. Sieber, H. J. Simonis, R. Ulrich, S. Wayand, M. Weber, T. Weiler, S. Williamson, C. Wöhrmann, R. Wolf, G. Anagnostou, G. Daskalakis, T. Geralis, A. Kyriakis, D. Loukas, I. Topsis-Giotis, G. Karathanasis, S. Kesisoglou, A. Panagiotou, N. Saoulidou, E. Tziaferi, K. Kousouris, I. Evangelou, C. Foudas, P. Gianneios, P. Katsoulis, P. Kokkas, S. Mallios, N. Manthos, I. Papadopoulos, E. Paradas, J. Strologas, F. A. Triantis, D. Tsitsonis, M. Csanad, N. Filipovic, G. Pasztor, O. Surányi, G. I. Veres, G. Bencze, C. Hajdu, D. Horvath, Á. Hunyadi, F. Sikler, V. Veszpremi, G. Vesztergombi, N. Beni, S. Czellar, J. Karancsi, A. Makovec, J. Molnar, Z. Szillasi, M. Bartók, P. Raics, Z. L. Trocsanyi, B. Ujvari, S. Choudhury, J. R. Komaragiri, S. Bahinipati, P. Mal, K. Mandal, A. Nayak, D. K. Sahoo, N. Sahoo, S. K. Swain, S. Bansal, S. B. Beri, V. Bhatnagar, R. Chawla, N. Dhingra, A. Kaur, M. Kaur, S. Kaur, R. Kumar, P. Kumari, A. Mehta, J. B. Singh, G. Walia, Ashok Kumar, Aashaq Shah, A. Bhardwaj, S. Chauhan, B. C. Choudhary, R. B. Garg, S. Keshri, A. Kumar, S. Malhotra, M. Naimuddin, K. Ranjan, R. Sharma, R. Bhardwaj, R. Bhattacharya, S. Bhattacharya, U. Bhawandeep, S. Dey, S. Dutt, S. Dutta, S. Ghosh, N. Majumdar, A. Modak, K. Mondal, S. Mukhopadhyay, S. Nandan, A. Purohit, A. Roy, S. Roy Chowdhury, S. Sarkar, M. Sharan, S. Thakur, P. K. Behera, R. Chudasama, D. Dutta, V. Jha, V. Kumar, A. K. Mohanty, P. K. Netrakanti, L. M. Pant, P. Shukla, A. Topkar, T. Aziz, S. Dugad, B. Mahakud, S. Mitra, G. B. Mohanty, N. Sur, B. Sutar, S. Banerjee, S. Bhattacharya, S. Chatterjee, P. Das, M. Guchait, Sa. Jain, S. Kumar, M. Maity, G. Majumder, K. Mazumdar, T. Sarkar, N. Wickramage, S. Chauhan, S. Dube, V. Hegde, A. Kapoor, K. Kothekar, S. Pandey, A. Rane, S. Sharma, S. Chenarani, E. Eskandari Tadavani, S. M. Etesami, M. Khakzad, M. Mohammadi Najafabadi, M. Naseri, S. Paktinat Mehdiabadi, F. Rezaei Hosseinabadi, B. Safarzadeh, M. Zeinali, M. Felcini, M. Grunewald, M. Abbrescia, C. Calabria, A. Colaleo, D. Creanza, L. Cristella, N. De Filippis, M. De Palma, F. Errico, L. Fiore, G. Iaselli, S. Lezki, G. Maggi, M. Maggi, G. Miniello, S. My, S. Nuzzo, A. Pompili, G. Pugliese, R. Radogna, A. Ranieri, G. Selvaggi, A. Sharma, L. Silvestris, R. Venditti, P. Verwilligen, G. Abbiendi, C. Battilana, D. Bonacorsi, L. Borgonovi, S. Braibant-Giacomelli, R. Campanini, P. Capiluppi, A. Castro, F. R. Cavallo, S. S. Chhibra, G. Codispoti, M. Cuffiani, G. M. Dallavalle, F. Fabbri, A. Fanfani, D. Fasanella, P. Giacomelli, C. Grandi, L. Guiducci, S. Marcellini, G. Masetti, A. Montanari, F. L. Navarria, A. Perrotta, A. M. Rossi, T. Rovelli, G. P. Siroli, N. Tosi, S. Albergo, S. Costa, A. Di Mattia, F. Giordano, R. Potenza, A. Tricomi, C. Tuve, G. Barbagli, K. Chatterjee, V. Ciulli, C. Civinini, R. D’Alessandro, E. Focardi, P. Lenzi, M. Meschini, S. Paoletti, L. Russo, G. Sguazzoni, D. Strom, L. Viliani, L. Benussi, S. Bianco, F. Fabbri, D. Piccolo, F. Primavera, V. Calvelli, F. Ferro, F. Ravera, E. Robutti, S. Tosi, A. Benaglia, A. Beschi, L. Brianza, F. Brivio, V. Ciriolo, M. E. Dinardo, S. Fiorendi, S. Gennai, A. Ghezzi, P. Govoni, M. Malberti, S. Malvezzi, R. A. Manzoni, D. Menasce, L. Moroni, M. Paganoni, D. Pedrini, S. Pigazzini, S. Ragazzi, T. Tabarelli de Fatis, S. Buontempo, N. Cavallo, S. Di Guida, F. Fabozzi, F. Fienga, A. O. M. Iorio, W. A. Khan, L. Lista, S. Meola, P. Paolucci, C. Sciacca, F. Thyssen, P. Azzi, N. Bacchetta, L. Benato, A. Boletti, R. Carlin, A. Carvalho Antunes De Oliveira, P. Checchia, M. Dall’Osso, P. De Castro Manzano, T. Dorigo, U. Dosselli, F. Gasparini, U. Gasparini, A. Gozzelino, S. Lacaprara, P. Lujan, M. Margoni, A. T. Meneguzzo, N. Pozzobon, P. Ronchese, R. Rossin, F. Simonetto, E. Torassa, M. Zanetti, P. Zotto, G. Zumerle, A. Braghieri, A. Magnani, P. Montagna, S. P. Ratti, V. Re, M. Ressegotti, C. Riccardi, P. Salvini, I. Vai, P. Vitulo, L. Alunni Solestizi, M. Biasini, G. M. Bilei, C. Cecchi, D. Ciangottini, L. Fanò, P. Lariccia, R. Leonardi, E. Manoni, G. Mantovani, V. Mariani, M. Menichelli, A. Rossi, A. Santocchia, D. Spiga, K. Androsov, P. Azzurri, G. Bagliesi, T. Boccali, L. Borrello, R. Castaldi, M. A. Ciocci, R. Dell’Orso, G. Fedi, L. Giannini, A. Giassi, M. T. Grippo, F. Ligabue, T. Lomtadze, E. Manca, G. Mandorli, A. Messineo, F. Palla, A. Rizzi, A. Savoy-Navarro, P. Spagnolo, R. Tenchini, G. Tonelli, A. Venturi, P. G. Verdini, L. Barone, F. Cavallari, M. Cipriani, N. Daci, D. Del Re, E. Di Marco, M. Diemoz, S. Gelli, E. Longo, F. Margaroli, B. Marzocchi, P. Meridiani, G. Organtini, R. Paramatti, F. Preiato, S. Rahatlou, C. Rovelli, F. Santanastasio, N. Amapane, R. Arcidiacono, S. Argiro, M. Arneodo, N. Bartosik, R. Bellan, C. Biino, N. Cartiglia, F. Cenna, M. Costa, R. Covarelli, A. Degano, N. Demaria, B. Kiani, C. Mariotti, S. Maselli, E. Migliore, V. Monaco, E. Monteil, M. Monteno, M. M. Obertino, L. Pacher, N. Pastrone, M. Pelliccioni, G. L. Pinna Angioni, A. Romero, M. Ruspa, R. Sacchi, K. Shchelina, V. Sola, A. Solano, A. Staiano, P. Traczyk, S. Belforte, M. Casarsa, F. Cossutti, G. Della Ricca, A. Zanetti, D. H. Kim, G. N. Kim, M. S. Kim, J. Lee, S. Lee, S. W. Lee, C. S. Moon, Y. D. Oh, S. Sekmen, D. C. Son, Y. C. Yang, H. Kim, D. H. Moon, G. Oh, J. A. Brochero Cifuentes, J. Goh, T. J. Kim, S. Cho, S. Choi, Y. Go, D. Gyun, S. Ha, B. Hong, Y. Jo, Y. Kim, K. Lee, K. S. Lee, S. Lee, J. Lim, S. K. Park, Y. Roh, J. Almond, J. Kim, J. S. Kim, H. Lee, K. Lee, K. Nam, S. B. Oh, B. C. Radburn-Smith, S. H. Seo, U. K. Yang, H. D. Yoo, G. B. Yu, H. Kim, J. H. Kim, J. S. H. Lee, I. C. Park, Y. Choi, C. Hwang, J. Lee, I. Yu, V. Dudenas, A. Juodagalvis, J. Vaitkus, I. Ahmed, Z. A. Ibrahim, M. A. B. Md Ali, F. Mohamad Idris, W. A. T. Wan Abdullah, M. N. Yusli, Z. Zolkapli, R. Reyes-Almanza, G. Ramirez-Sanchez, M. C. Duran-Osuna, H. Castilla-Valdez, E. De La Cruz-Burelo, I. Heredia-De La Cruz, R. I. Rabadan-Trejo, R. Lopez-Fernandez, J. Mejia Guisao, A. Sanchez-Hernandez, S. Carrillo Moreno, C. Oropeza Barrera, F. Vazquez Valencia, J. Eysermans, I. Pedraza, H. A. Salazar Ibarguen, C. Uribe Estrada, A. Morelos Pineda, D. Krofcheck, P. H. Butler, A. Ahmad, M. Ahmad, Q. Hassan, H. R. Hoorani, A. Saddique, M. A. Shah, M. Shoaib, M. Waqas, H. Bialkowska, M. Bluj, B. Boimska, T. Frueboes, M. Górski, M. Kazana, K. Nawrocki, M. Szleper, P. Zalewski, K. Bunkowski, A. Byszuk, K. Doroba, A. Kalinowski, M. Konecki, J. Krolikowski, M. Misiura, M. Olszewski, A. Pyskir, M. Walczak, P. Bargassa, C. Beirão Da Cruz E. Silva, A. Di Francesco, P. Faccioli, B. Galinhas, M. Gallinaro, J. Hollar, N. Leonardo, L. Lloret Iglesias, M. V. Nemallapudi, J. Seixas, G. Strong, O. Toldaiev, D. Vadruccio, J. Varela, A. Baginyan, A. Golunov, I. Golutvin, V. Karjavin, V. Korenkov, G. Kozlov, A. Lanev, A. Malakhov, V. Matveev, V. V. Mitsyn, P. Moisenz, V. Palichik, V. Perelygin, S. Shmatov, V. Smirnov, N. Voytishin, B. S. Yuldashev, A. Zarubin, V. Zhiltsov, Y. Ivanov, V. Kim, E. Kuznetsova, P. Levchenko, V. Murzin, V. Oreshkin, I. Smirnov, D. Sosnov, V. Sulimov, L. Uvarov, S. Vavilov, A. Vorobyev, Yu. Andreev, A. Dermenev, S. Gninenko, N. Golubev, A. Karneyeu, M. Kirsanov, N. Krasnikov, A. Pashenkov, D. Tlisov, A. Toropin, V. Epshteyn, V. Gavrilov, N. Lychkovskaya, V. Popov, I. Pozdnyakov, G. Safronov, A. Spiridonov, A. Stepennov, V. Stolin, M. Toms, E. Vlasov, A. Zhokin, T. Aushev, A. Bylinkin, R. Chistov, M. Danilov, P. Parygin, D. Philippov, S. Polikarpov, E. Tarkovskii, V. Andreev, M. Azarkin, I. Dremin, M. Kirakosyan, S. V. Rusakov, A. Terkulov, A. Baskakov, A. Belyaev, E. Boos, V. Bunichev, M. Dubinin, L. Dudko, A. Gribushin, V. Klyukhin, N. Korneeva, I. Lokhtin, I. Miagkov, S. Obraztsov, M. Perfilov, V. Savrin, P. Volkov, V. Blinov, D. Shtol, Y. Skovpen, I. Azhgirey, I. Bayshev, S. Bitioukov, D. Elumakhov, A. Godizov, V. Kachanov, A. Kalinin, D. Konstantinov, P. Mandrik, V. Petrov, R. Ryutin, A. Sobol, S. Troshin, N. Tyurin, A. Uzunian, A. Volkov, P. Adzic, P. Cirkovic, D. Devetak, M. Dordevic, J. Milosevic, J. Alcaraz Maestre, I. Bachiller, M. Barrio Luna, M. Cerrada, N. Colino, B. De La Cruz, A. Delgado Peris, C. Fernandez Bedoya, J. P. Fernández Ramos, J. Flix, M. C. Fouz, O. Gonzalez Lopez, S. Goy Lopez, J. M. Hernandez, M. I. Josa, D. Moran, A. Pérez-Calero Yzquierdo, J. Puerta Pelayo, I. Redondo, L. Romero, M. S. Soares, A. Triossi, A. Álvarez Fernández, C. Albajar, J. F. de Trocóniz, J. Cuevas, C. Erice, J. Fernandez Menendez, I. Gonzalez Caballero, J. R. González Fernández, E. Palencia Cortezon, S. Sanchez Cruz, P. Vischia, J. M. Vizan Garcia, I. J. Cabrillo, A. Calderon, B. Chazin Quero, E. Curras, J. Duarte Campderros, M. Fernandez, J. Garcia-Ferrero, G. Gomez, A. Lopez Virto, J. Marco, C. Martinez Rivero, P. Martinez Ruiz del Arbol, F. Matorras, J. Piedra Gomez, T. Rodrigo, A. Ruiz-Jimeno, L. Scodellaro, N. Trevisani, I. Vila, R. Vilar Cortabitarte, D. Abbaneo, B. Akgun, E. Auffray, P. Baillon, A. H. Ball, D. Barney, J. Bendavid, M. Bianco, A. Bocci, C. Botta, T. Camporesi, R. Castello, M. Cepeda, G. Cerminara, E. Chapon, Y. Chen, D. d’Enterria, A. Dabrowski, V. Daponte, A. David, M. De Gruttola, A. De Roeck, N. Deelen, M. Dobson, T. du Pree, M. Dünser, N. Dupont, A. Elliott-Peisert, P. Everaerts, F. Fallavollita, G. Franzoni, J. Fulcher, W. Funk, D. Gigi, A. Gilbert, K. Gill, F. Glege, D. Gulhan, P. Harris, J. Hegeman, V. Innocente, A. Jafari, P. Janot, O. Karacheban, J. Kieseler, V. Knünz, A. Kornmayer, M. J. Kortelainen, M. Krammer, C. Lange, P. Lecoq, C. Lourenço, M. T. Lucchini, L. Malgeri, M. Mannelli, A. Martelli, F. Meijers, J. A. Merlin, S. Mersi, E. Meschi, P. Milenovic, F. Moortgat, M. Mulders, H. Neugebauer, J. Ngadiuba, S. Orfanelli, L. Orsini, L. Pape, E. Perez, M. Peruzzi, A. Petrilli, G. Petrucciani, A. Pfeiffer, M. Pierini, D. Rabady, A. Racz, T. Reis, G. Rolandi, M. Rovere, H. Sakulin, C. Schäfer, C. Schwick, M. Seidel, M. Selvaggi, A. Sharma, P. Silva, P. Sphicas, A. Stakia, J. Steggemann, M. Stoye, M. Tosi, D. Treille, A. Tsirou, V. Veckalns, M. Verweij, W. D. Zeuner, W. Bertl, L. Caminada, K. Deiters, W. Erdmann, R. Horisberger, Q. Ingram, H. C. Kaestli, D. Kotlinski, U. Langenegger, T. Rohe, S. A. Wiederkehr, M. Backhaus, L. Bäni, P. Berger, L. Bianchini, B. Casal, G. Dissertori, M. Dittmar, M. Donegà, C. Dorfer, C. Grab, C. Heidegger, D. Hits, J. Hoss, G. Kasieczka, T. Klijnsma, W. Lustermann, B. Mangano, M. Marionneau, M. T. Meinhard, D. Meister, F. Micheli, P. Musella, F. Nessi-Tedaldi, F. Pandolfi, J. Pata, F. Pauss, G. Perrin, L. Perrozzi, M. Quittnat, M. Reichmann, D. A. Sanz Becerra, M. Schönenberger, L. Shchutska, V. R. Tavolaro, K. Theofilatos, M. L. Vesterbacka Olsson, R. Wallny, D. H. Zhu, T. K. Aarrestad, C. Amsler, M. F. Canelli, A. De Cosa, R. Del Burgo, S. Donato, C. Galloni, T. Hreus, B. Kilminster, D. Pinna, G. Rauco, P. Robmann, D. Salerno, K. Schweiger, C. Seitz, Y. Takahashi, A. Zucchetta, V. Candelise, Y. H. Chang, K. y. Cheng, T. H. Doan, Sh. Jain, R. Khurana, C. M. Kuo, W. Lin, A. Pozdnyakov, S. S. Yu, Arun Kumar, P. Chang, Y. Chao, K. F. Chen, P. H. Chen, F. Fiori, W.-S. Hou, Y. Hsiung, Y. F. Liu, R.-S. Lu, E. Paganis, A. Psallidas, A. Steen, J. F. Tsai, B. Asavapibhop, K. Kovitanggoon, G. Singh, N. Srimanobhas, A. Bat, F. Boran, S. Damarseckin, Z. S. Demiroglu, C. Dozen, E. Eskut, S. Girgis, G. Gokbulut, Y. Guler, I. Hos, E. E. Kangal, O. Kara, A. Kayis Topaksu, U. Kiminsu, M. Oglakci, G. Onengut, K. Ozdemir, S. Ozturk, A. Polatoz, U. G. Tok, H. Topakli, B. Tali, S. Turkcapar, I. S. Zorbakir, C. Zorbilmez, G. Karapinar, K. Ocalan, M. Yalvac, M. Zeyrek, E. Gülmez, M. Kaya, O. Kaya, S. Tekten, E. A. Yetkin, M. N. Agaras, S. Atay, A. Cakir, K. Cankocak, Y. Komurcu, B. Grynyov, L. Levchuk, F. Ball, L. Beck, J. J. Brooke, D. Burns, E. Clement, D. Cussans, O. Davignon, H. Flacher, J. Goldstein, G. P. Heath, H. F. Heath, L. Kreczko, D. M. Newbold, S. Paramesvaran, T. Sakuma, S. Seif El Nasr-storey, D. Smith, V. J. Smith, K. W. Bell, A. Belyaev, C. Brew, R. M. Brown, L. Calligaris, D. Cieri, D. J. A. Cockerill, J. A. Coughlan, K. Harder, S. Harper, J. Linacre, E. Olaiya, D. Petyt, C. H. Shepherd-Themistocleous, A. Thea, I. R. Tomalin, T. Williams, W. J. Womersley, G. Auzinger, R. Bainbridge, P. Bloch, J. Borg, S. Breeze, O. Buchmuller, A. Bundock, S. Casasso, M. Citron, D. Colling, L. Corpe, P. Dauncey, G. Davies, A. De Wit, M. Della Negra, R. Di Maria, A. Elwood, Y. Haddad, G. Hall, G. Iles, T. James, R. Lane, C. Laner, L. Lyons, A.-M. Magnan, S. Malik, L. Mastrolorenzo, T. Matsushita, J. Nash, A. Nikitenko, V. Palladino, M. Pesaresi, D. M. Raymond, A. Richards, A. Rose, E. Scott, C. Seez, A. Shtipliyski, S. Summers, A. Tapper, K. Uchida, M. Vazquez Acosta, T. Virdee, N. Wardle, D. Winterbottom, J. Wright, S. C. Zenz, J. E. Cole, P. R. Hobson, A. Khan, P. Kyberd, I. D. Reid, L. Teodorescu, S. Zahid, A. Borzou, K. Call, J. Dittmann, K. Hatakeyama, H. Liu, N. Pastika, C. Smith, R. Bartek, A. Dominguez, A. Buccilli, S. I. Cooper, C. Henderson, P. Rumerio, C. West, D. Arcaro, A. Avetisyan, T. Bose, D. Gastler, D. Rankin, C. Richardson, J. Rohlf, L. Sulak, D. Zou, G. Benelli, D. Cutts, M. Hadley, J. Hakala, U. Heintz, J. M. Hogan, K. H. M. Kwok, E. Laird, G. Landsberg, J. Lee, Z. Mao, M. Narain, J. Pazzini, S. Piperov, S. Sagir, R. Syarif, D. Yu, R. Band, C. Brainerd, R. Breedon, D. Burns, M. Calderon De La Barca Sanchez, M. Chertok, J. Conway, R. Conway, P. T. Cox, R. Erbacher, C. Flores, G. Funk, W. Ko, R. Lander, C. Mclean, M. Mulhearn, D. Pellett, J. Pilot, S. Shalhout, M. Shi, J. Smith, D. Stolp, K. Tos, M. Tripathi, Z. Wang, M. Bachtis, C. Bravo, R. Cousins, A. Dasgupta, A. Florent, J. Hauser, M. Ignatenko, N. Mccoll, S. Regnard, D. Saltzberg, C. Schnaible, V. Valuev, E. Bouvier, K. Burt, R. Clare, J. Ellison, J. W. Gary, S. M. A. Ghiasi Shirazi, G. Hanson, J. Heilman, G. Karapostoli, E. Kennedy, F. Lacroix, O. R. Long, M. Olmedo Negrete, M. I. Paneva, W. Si, L. Wang, H. Wei, S. Wimpenny, B. R. Yates, J. G. Branson, S. Cittolin, M. Derdzinski, R. Gerosa, D. Gilbert, B. Hashemi, A. Holzner, D. Klein, G. Kole, V. Krutelyov, J. Letts, M. Masciovecchio, D. Olivito, S. Padhi, M. Pieri, M. Sani, V. Sharma, S. Simon, M. Tadel, A. Vartak, S. Wasserbaech, J. Wood, F. Würthwein, A. Yagil, G. Zevi Della Porta, N. Amin, R. Bhandari, J. Bradmiller-Feld, C. Campagnari, A. Dishaw, V. Dutta, M. Franco Sevilla, L. Gouskos, R. Heller, J. Incandela, A. Ovcharova, H. Qu, J. Richman, D. Stuart, I. Suarez, J. Yoo, D. Anderson, A. Bornheim, J. Bunn, J. M. Lawhorn, H. B. Newman, T. Q. Nguyen, C. Pena, M. Spiropulu, J. R. Vlimant, R. Wilkinson, S. Xie, Z. Zhang, R. Y. Zhu, M. B. Andrews, T. Ferguson, T. Mudholkar, M. Paulini, J. Russ, M. Sun, H. Vogel, I. Vorobiev, M. Weinberg, J. P. Cumalat, W. T. Ford, F. Jensen, A. Johnson, M. Krohn, S. Leontsinis, T. Mulholland, K. Stenson, K. A. Ulmer, S. R. Wagner, J. Alexander, J. Chaves, J. Chu, S. Dittmer, K. Mcdermott, N. Mirman, J. R. Patterson, D. Quach, A. Rinkevicius, A. Ryd, L. Skinnari, L. Soffi, S. M. Tan, Z. Tao, J. Thom, J. Tucker, P. Wittich, M. Zientek, S. Abdullin, M. Albrow, M. Alyari, G. Apollinari, A. Apresyan, A. Apyan, S. Banerjee, L. A. T. Bauerdick, A. Beretvas, J. Berryhill, P. C. Bhat, G. Bolla, K. Burkett, J. N. Butler, A. Canepa, G. B. Cerati, H. W. K. Cheung, F. Chlebana, M. Cremonesi, J. Duarte, V. D. Elvira, J. Freeman, Z. Gecse, E. Gottschalk, L. Gray, D. Green, S. Grünendahl, O. Gutsche, J. Hanlon, R. M. Harris, S. Hasegawa, J. Hirschauer, Z. Hu, B. Jayatilaka, S. Jindariani, M. Johnson, U. Joshi, B. Klima, B. Kreis, S. Lammel, D. Lincoln, R. Lipton, M. Liu, T. Liu, R. Lopes De Sá, J. Lykken, K. Maeshima, N. Magini, J. M. Marraffino, D. Mason, P. McBride, P. Merkel, S. Mrenna, S. Nahn, V. O’Dell, K. Pedro, O. Prokofyev, G. Rakness, L. Ristori, B. Schneider, E. Sexton-Kennedy, A. Soha, W. J. Spalding, L. Spiegel, S. Stoynev, J. Strait, N. Strobbe, L. Taylor, S. Tkaczyk, N. V. Tran, L. Uplegger, E. W. Vaandering, C. Vernieri, M. Verzocchi, R. Vidal, M. Wang, H. A. Weber, A. Whitbeck, W. Wu, D. Acosta, P. Avery, P. Bortignon, D. Bourilkov, A. Brinkerhoff, A. Carnes, M. Carver, D. Curry, R. D. Field, I. K. Furic, S. V. Gleyzer, B. M. Joshi, J. Konigsberg, A. Korytov, K. Kotov, P. Ma, K. Matchev, H. Mei, G. Mitselmakher, K. Shi, D. Sperka, N. Terentyev, L. Thomas, J. Wang, S. Wang, J. Yelton, Y. R. Joshi, S. Linn, P. Markowitz, J. L. Rodriguez, A. Ackert, T. Adams, A. Askew, S. Hagopian, V. Hagopian, K. F. Johnson, T. Kolberg, G. Martinez, T. Perry, H. Prosper, A. Saha, A. Santra, V. Sharma, R. Yohay, M. M. Baarmand, V. Bhopatkar, S. Colafranceschi, M. Hohlmann, D. Noonan, T. Roy, F. Yumiceva, M. R. Adams, L. Apanasevich, D. Berry, R. R. Betts, R. Cavanaugh, X. Chen, O. Evdokimov, C. E. Gerber, D. A. Hangal, D. J. Hofman, K. Jung, J. Kamin, I. D. Sandoval Gonzalez, M. B. Tonjes, H. Trauger, N. Varelas, H. Wang, Z. Wu, J. Zhang, B. Bilki, W. Clarida, K. Dilsiz, S. Durgut, R. P. Gandrajula, M. Haytmyradov, V. Khristenko, J.-P. Merlo, H. Mermerkaya, A. Mestvirishvili, A. Moeller, J. Nachtman, H. Ogul, Y. Onel, F. Ozok, A. Penzo, C. Snyder, E. Tiras, J. Wetzel, K. Yi, B. Blumenfeld, A. Cocoros, N. Eminizer, D. Fehling, L. Feng, A. V. Gritsan, P. Maksimovic, J. Roskes, U. Sarica, M. Swartz, M. Xiao, C. You, A. Al-bataineh, P. Baringer, A. Bean, S. Boren, J. Bowen, J. Castle, S. Khalil, A. Kropivnitskaya, D. Majumder, W. Mcbrayer, M. Murray, C. Rogan, C. Royon, S. Sanders, E. Schmitz, J. D. Tapia Takaki, Q. Wang, A. Ivanov, K. Kaadze, Y. Maravin, A. Mohammadi, L. K. Saini, N. Skhirtladze, F. Rebassoo, D. Wright, A. Baden, O. Baron, A. Belloni, S. C. Eno, Y. Feng, C. Ferraioli, N. J. Hadley, S. Jabeen, G. Y. Jeng, R. G. Kellogg, J. Kunkle, A. C. Mignerey, F. Ricci-Tam, Y. H. Shin, A. Skuja, S. C. Tonwar, D. Abercrombie, B. Allen, V. Azzolini, R. Barbieri, A. Baty, G. Bauer, R. Bi, S. Brandt, W. Busza, I. A. Cali, M. D’Alfonso, Z. Demiragli, G. Gomez Ceballos, M. Goncharov, D. Hsu, M. Hu, Y. Iiyama, G. M. Innocenti, M. Klute, D. Kovalskyi, Y.-J. Lee, A. Levin, P. D. Luckey, B. Maier, A. C. Marini, C. Mcginn, C. Mironov, S. Narayanan, X. Niu, C. Paus, C. Roland, G. Roland, J. Salfeld-Nebgen, G. S. F. Stephans, K. Sumorok, K. Tatar, D. Velicanu, J. Wang, T. W. Wang, B. Wyslouch, A. C. Benvenuti, R. M. Chatterjee, A. Evans, P. Hansen, J. Hiltbrand, S. Kalafut, Y. Kubota, Z. Lesko, J. Mans, S. Nourbakhsh, N. Ruckstuhl, R. Rusack, J. Turkewitz, M. A. Wadud, J. G. Acosta, S. Oliveros, E. Avdeeva, K. Bloom, D. R. Claes, C. Fangmeier, F. Golf, R. Gonzalez Suarez, R. Kamalieddin, I. Kravchenko, J. Monroy, J. E. Siado, G. R. Snow, B. Stieger, J. Dolen, A. Godshalk, C. Harrington, I. Iashvili, D. Nguyen, A. Parker, S. Rappoccio, B. Roozbahani, G. Alverson, E. Barberis, C. Freer, A. Hortiangtham, A. Massironi, D. M. Morse, T. Orimoto, R. Teixeira De Lima, T. Wamorkar, B. Wang, A. Wisecarver, D. Wood, S. Bhattacharya, O. Charaf, K. A. Hahn, N. Mucia, N. Odell, M. H. Schmitt, K. Sung, M. Trovato, M. Velasco, R. Bucci, N. Dev, M. Hildreth, K. Hurtado Anampa, C. Jessop, D. J. Karmgard, N. Kellams, K. Lannon, W. Li, N. Loukas, N. Marinelli, F. Meng, C. Mueller, Y. Musienko, M. Planer, A. Reinsvold, R. Ruchti, P. Siddireddy, G. Smith, S. Taroni, M. Wayne, A. Wightman, M. Wolf, A. Woodard, J. Alimena, L. Antonelli, B. Bylsma, L. S. Durkin, S. Flowers, B. Francis, A. Hart, C. Hill, W. Ji, T. Y. Ling, B. Liu, W. Luo, B. L. Winer, H. W. Wulsin, S. Cooperstein, O. Driga, P. Elmer, J. Hardenbrook, P. Hebda, S. Higginbotham, A. Kalogeropoulos, D. Lange, J. Luo, D. Marlow, K. Mei, I. Ojalvo, J. Olsen, C. Palmer, P. Piroué, D. Stickland, C. Tully, S. Malik, S. Norberg, A. Barker, V. E. Barnes, S. Das, S. Folgueras, L. Gutay, M. Jones, A. W. Jung, A. Khatiwada, D. H. Miller, N. Neumeister, C. C. Peng, H. Qiu, J. F. Schulte, J. Sun, F. Wang, R. Xiao, W. Xie, T. Cheng, N. Parashar, J. Stupak, Z. Chen, K. M. Ecklund, S. Freed, F. J. M. Geurts, M. Guilbaud, M. Kilpatrick, W. Li, B. Michlin, B. P. Padley, J. Roberts, J. Rorie, W. Shi, Z. Tu, J. Zabel, A. Zhang, A. Bodek, P. de Barbaro, R. Demina, Y. T. Duh, T. Ferbel, M. Galanti, A. Garcia-Bellido, J. Han, O. Hindrichs, A. Khukhunaishvili, K. H. Lo, P. Tan, M. Verzetti, R. Ciesielski, K. Goulianos, C. Mesropian, A. Agapitos, J. P. Chou, Y. Gershtein, T. A. Gómez Espinosa, E. Halkiadakis, M. Heindl, E. Hughes, S. Kaplan, R. Kunnawalkam Elayavalli, S. Kyriacou, A. Lath, R. Montalvo, K. Nash, M. Osherson, H. Saka, S. Salur, S. Schnetzer, D. Sheffield, S. Somalwar, R. Stone, S. Thomas, P. Thomassen, M. Walker, A. G. Delannoy, J. Heideman, G. Riley, K. Rose, S. Spanier, K. Thapa, O. Bouhali, A. Castaneda Hernandez, A. Celik, M. Dalchenko, M. De Mattia, A. Delgado, S. Dildick, R. Eusebi, J. Gilmore, T. Huang, T. Kamon, R. Mueller, Y. Pakhotin, R. Patel, A. Perloff, L. Perniè, D. Rathjens, A. Safonov, A. Tatarinov, N. Akchurin, J. Damgov, F. De Guio, P. R. Dudero, J. Faulkner, E. Gurpinar, S. Kunori, K. Lamichhane, S. W. Lee, T. Libeiro, T. Mengke, S. Muthumuni, T. Peltola, S. Undleeb, I. Volobouev, Z. Wang, S. Greene, A. Gurrola, R. Janjam, W. Johns, C. Maguire, A. Melo, H. Ni, K. Padeken, P. Sheldon, S. Tuo, J. Velkovska, Q. Xu, M. W. Arenton, P. Barria, B. Cox, R. Hirosky, M. Joyce, A. Ledovskoy, H. Li, C. Neu, T. Sinthuprasith, Y. Wang, E. Wolfe, F. Xia, R. Harr, P. E. Karchin, N. Poudyal, J. Sturdy, P. Thapa, S. Zaleski, M. Brodski, J. Buchanan, C. Caillol, D. Carlsmith, S. Dasu, L. Dodd, S. Duric, B. Gomber, M. Grothe, M. Herndon, A. Hervé, U. Hussain, P. Klabbers, A. Lanaro, A. Levine, K. Long, R. Loveless, V. Rekovic, T. Ruggles, A. Savin, N. Smith, W. H. Smith, D. Taylor, N. Woods, CMS Collaboration

**Affiliations:** 1 Yerevan Physics Institute, Yerevan, Armenia; 2 Institut für Hochenergiephysik, Vienna, Austria; 3 Institute for Nuclear Problems, Minsk, Belarus; 4 Universiteit Antwerpen, Antwerp, Belgium; 5 Vrije Universiteit Brussel, Brussels, Belgium; 6 Université Libre de Bruxelles, Brussels, Belgium; 7 Ghent University, Ghent, Belgium; 8 Université Catholique de Louvain, Louvain-la-Neuve, Belgium; 9 Centro Brasileiro de Pesquisas Fisicas, Rio de Janeiro, Brazil; 10 Universidade do Estado do Rio de Janeiro, Rio de Janeiro, Brazil; 11 Universidade Estadual Paulista, Universidade Federal do ABC, São Paulo, Brazil; 12 Institute for Nuclear Research and Nuclear Energy, Bulgarian Academy of Sciences, Sofia, Bulgaria; 13 University of Sofia, Sofia, Bulgaria; 14 Beihang University, Beijing, China; 15 Institute of High Energy Physics, Beijing, China; 16 State Key Laboratory of Nuclear Physics and Technology, Peking University, Beijing, China; 17 Tsinghua University, Beijing, China; 18 Universidad de Los Andes, Bogotá, Colombia; 19 Faculty of Electrical Engineering, Mechanical Engineering and Naval Architecture, University of Split, Split, Croatia; 20 Faculty of Science, University of Split, Split, Croatia; 21 Institute Rudjer Boskovic, Zagreb, Croatia; 22 University of Cyprus, Nicosia, Cyprus; 23 Charles University, Prague, Czech Republic; 24 Universidad San Francisco de Quito, Quito, Ecuador; 25 Academy of Scientific Research and Technology of the Arab Republic of Egypt, Egyptian Network of High Energy Physics, Cairo, Egypt; 26 National Institute of Chemical Physics and Biophysics, Tallinn, Estonia; 27 Department of Physics, University of Helsinki, Helsinki, Finland; 28 Helsinki Institute of Physics, Helsinki, Finland; 29 Lappeenranta University of Technology, Lappeenranta, Finland; 30 IRFU, CEA, Université Paris-Saclay, Gif-sur-Yvette, France; 31 Laboratoire Leprince-Ringuet, Ecole Polytechnique, CNRS/IN2P3, Université Paris-Saclay, Palaiseau, France; 32 Université de Strasbourg, CNRS, IPHC UMR 7178, 67000, Strasbourg, France; 33 Centre de Calcul de l’Institut National de Physique Nucleaire et de Physique des Particules, CNRS/IN2P3, Villeurbanne, France; 34 Université de Lyon, Université Claude Bernard Lyon 1, CNRS-IN2P3, Institut de Physique Nucléaire de Lyon, Villeurbanne, France; 35 Georgian Technical University, Tbilisi, Georgia; 36 Tbilisi State University, Tbilisi, Georgia; 37 RWTH Aachen University, I. Physikalisches Institut, Aachen, Germany; 38 RWTH Aachen University, III. Physikalisches Institut A, Aachen, Germany; 39 RWTH Aachen University, III. Physikalisches Institut B, Aachen, Germany; 40 Deutsches Elektronen-Synchrotron, Hamburg, Germany; 41 University of Hamburg, Hamburg, Germany; 42 Institut für Experimentelle Kernphysik, Karlsruhe, Germany; 43 Institute of Nuclear and Particle Physics (INPP), NCSR Demokritos, Aghia Paraskevi, Greece; 44 National and Kapodistrian University of Athens, Athens, Greece; 45 National Technical University of Athens, Athens, Greece; 46 University of Ioánnina, Ioannina, Greece; 47 MTA-ELTE Lendület CMS Particle and Nuclear Physics Group, Eötvös Loránd University, Budapest, Hungary; 48 Wigner Research Centre for Physics, Budapest, Hungary; 49 Institute of Nuclear Research ATOMKI, Debrecen, Hungary; 50 Institute of Physics, University of Debrecen, Debrecen, Hungary; 51 Indian Institute of Science (IISc), Bangalore, India; 52 National Institute of Science Education and Research, Bhubaneswar, India; 53 Panjab University, Chandigarh, India; 54 University of Delhi, Delhi, India; 55 Saha Institute of Nuclear Physics, HBNI, Kolkata, India; 56 Indian Institute of Technology Madras, Madras, India; 57 Bhabha Atomic Research Centre, Mumbai, India; 58 Tata Institute of Fundamental Research-A, Mumbai, India; 59 Tata Institute of Fundamental Research-B, Mumbai, India; 60 Indian Institute of Science Education and Research (IISER), Pune, India; 61 Institute for Research in Fundamental Sciences (IPM), Tehran, Iran; 62 University College Dublin, Dublin, Ireland; 63 INFN Sezione di Bari, Università di Bari, Politecnico di Bari, Bari, Italy; 64 INFN Sezione di Bologna, Università di Bologna, Bologna, Italy; 65 INFN Sezione di Catania, Università di Catania, Catania, Italy; 66 INFN Sezione di Firenze, Università di Firenze, Florence, Italy; 67 INFN Laboratori Nazionali di Frascati, Frascati, Italy; 68 INFN Sezione di Genova, Università di Genova, Genoa, Italy; 69 INFN Sezione di Milano-Bicocca, Università di Milano-Bicocca, Milan, Italy; 70 INFN Sezione di Napoli, Università di Napoli ‘Federico II’, Naples, Italy, Università della Basilicata, Potenza, Italy, Università G. Marconi, Rome, Italy; 71 INFN Sezione di Padova, Università di Padova, Padua, Italy, Università di Trento, Trento, Italy; 72 INFN Sezione di Pavia, Università di Pavia, Pavia, Italy; 73 INFN Sezione di Perugia, Università di Perugia, Perugia, Italy; 74 INFN Sezione di Pisa, Università di Pisa, Scuola Normale Superiore di Pisa, Pisa, Italy; 75 INFN Sezione di Roma, Università di Roma, Rome, Italy; 76 INFN Sezione di Torino, Università di Torino, Turin, Italy, Università del Piemonte Orientale, Novara, Italy; 77 INFN Sezione di Trieste, Università di Trieste, Trieste, Italy; 78 Kyungpook National University, Daegu, Korea; 79 Institute for Universe and Elementary Particles, Chonnam National University, Kwangju, Korea; 80 Hanyang University, Seoul, Korea; 81 Korea University, Seoul, Korea; 82 Seoul National University, Seoul, Korea; 83 University of Seoul, Seoul, Korea; 84 Sungkyunkwan University, Suwon, Korea; 85 Vilnius University, Vilnius, Lithuania; 86 National Centre for Particle Physics, Universiti Malaya, Kuala Lumpur, Malaysia; 87 Centro de Investigacion y de Estudios Avanzados del IPN, Mexico City, Mexico; 88 Universidad Iberoamericana, Mexico City, Mexico; 89 Benemerita Universidad Autonoma de Puebla, Puebla, Mexico; 90 Universidad Autónoma de San Luis Potosí, San Luis Potosí, Mexico; 91 University of Auckland, Auckland, New Zealand; 92 University of Canterbury, Christchurch, New Zealand; 93 National Centre for Physics, Quaid-I-Azam University, Islamabad, Pakistan; 94 National Centre for Nuclear Research, Swierk, Poland; 95 Faculty of Physics, Institute of Experimental Physics, University of Warsaw, Warsaw, Poland; 96 Laboratório de Instrumentação e Física Experimental de Partículas, Lisbon, Portugal; 97 Joint Institute for Nuclear Research, Dubna, Russia; 98 Petersburg Nuclear Physics Institute, Gatchina (St. Petersburg), Russia; 99 Institute for Nuclear Research, Moscow, Russia; 100 Institute for Theoretical and Experimental Physics, Moscow, Russia; 101 Moscow Institute of Physics and Technology, Moscow, Russia; 102 National Research Nuclear University ‘Moscow Engineering Physics Institute’ (MEPhI), Moscow, Russia; 103 P.N. Lebedev Physical Institute, Moscow, Russia; 104 Skobeltsyn Institute of Nuclear Physics, Lomonosov Moscow State University, Moscow, Russia; 105 Novosibirsk State University (NSU), Novosibirsk, Russia; 106 State Research Center of Russian Federation, Institute for High Energy Physics of NRC “Kurchatov Institute”, Protvino, Russia; 107 Faculty of Physics and Vinca Institute of Nuclear Sciences, University of Belgrade, Belgrade, Serbia; 108 Centro de Investigaciones Energéticas Medioambientales y Tecnológicas (CIEMAT), Madrid, Spain; 109 Universidad Autónoma de Madrid, Madrid, Spain; 110 Universidad de Oviedo, Oviedo, Spain; 111 Instituto de Física de Cantabria (IFCA), CSIC-Universidad de Cantabria, Santander, Spain; 112 CERN, European Organization for Nuclear Research, Geneva, Switzerland; 113 Paul Scherrer Institut, Villigen, Switzerland; 114 Institute for Particle Physics and Astrophysics (IPA), ETH Zurich, Zurich, Switzerland; 115 Universität Zürich, Zurich, Switzerland; 116 National Central University, Chung-Li, Taiwan; 117 National Taiwan University (NTU), Taipei, Taiwan; 118 Department of Physics, Faculty of Science, Chulalongkorn University, Bangkok, Thailand; 119 Physics Department, Science and Art Faculty, Çukurova University, Adana, Turkey; 120 Physics Department, Middle East Technical University, Ankara, Turkey; 121 Bogazici University, Istanbul, Turkey; 122 Istanbul Technical University, Istanbul, Turkey; 123 Institute for Scintillation Materials of National Academy of Science of Ukraine, Kharkov, Ukraine; 124 National Scientific Center, Kharkov Institute of Physics and Technology, Kharkov, Ukraine; 125 University of Bristol, Bristol, UK; 126 Rutherford Appleton Laboratory, Didcot, UK; 127 Imperial College, London, UK; 128 Brunel University, Uxbridge, UK; 129 Baylor University, Waco, USA; 130 Catholic University of America, Washington, DC, USA; 131 The University of Alabama, Tuscaloosa, USA; 132 Boston University, Boston, USA; 133 Brown University, Providence, USA; 134 University of California, Davis, Davis, USA; 135 University of California, Los Angeles, USA; 136 University of California, Riverside, Riverside, USA; 137 University of California, San Diego, La Jolla, USA; 138 Santa Barbara-Department of Physics, University of California, Santa Barbara, USA; 139 California Institute of Technology, Pasadena, USA; 140 Carnegie Mellon University, Pittsburgh, USA; 141 University of Colorado Boulder, Boulder, USA; 142 Cornell University, Ithaca, USA; 143 Fermi National Accelerator Laboratory, Batavia, USA; 144 University of Florida, Gainesville, USA; 145 Florida International University, Miami, USA; 146 Florida State University, Tallahassee, USA; 147 Florida Institute of Technology, Melbourne, USA; 148 University of Illinois at Chicago (UIC), Chicago, USA; 149 The University of Iowa, Iowa City, USA; 150 Johns Hopkins University, Baltimore, USA; 151 The University of Kansas, Lawrence, USA; 152 Kansas State University, Manhattan, USA; 153 Lawrence Livermore National Laboratory, Livermore, USA; 154 University of Maryland, College Park, USA; 155 Massachusetts Institute of Technology, Cambridge, USA; 156 University of Minnesota, Minneapolis, USA; 157 University of Mississippi, Oxford, USA; 158 University of Nebraska-Lincoln, Lincoln, USA; 159 State University of New York at Buffalo, Buffalo, USA; 160 Northeastern University, Boston, USA; 161 Northwestern University, Evanston, USA; 162 University of Notre Dame, Notre Dame, USA; 163 The Ohio State University, Columbus, USA; 164 Princeton University, Princeton, USA; 165 University of Puerto Rico, Mayagüez, USA; 166 Purdue University, West Lafayette, USA; 167 Purdue University Northwest, Hammond, USA; 168 Rice University, Houston, USA; 169 University of Rochester, Rochester, USA; 170 The Rockefeller University, New York, USA; 171 Rutgers, The State University of New Jersey, Piscataway, USA; 172 University of Tennessee, Knoxville, USA; 173 Texas A&M University, College Station, USA; 174 Texas Tech University, Lubbock, USA; 175 Vanderbilt University, Nashville, USA; 176 University of Virginia, Charlottesville, USA; 177 Wayne State University, Detroit, USA; 178 University of Wisconsin-Madison, Madison, WI USA; 179 CERN, 1211 Geneva 23, Switzerland

## Abstract

A search for standard model production of four top quarks ($\mathrm {t}\overline{\mathrm {t}} \mathrm {t}\overline{\mathrm {t}}$) is reported using events containing at least three leptons ($\mathrm {e}, \mathrm {\mu }$) or a same-sign lepton pair. The events are produced in proton–proton collisions at a center-of-mass energy of 13$\,\text {TeV}$ at the LHC, and the data sample, recorded in 2016, corresponds to an integrated luminosity of 35.9$\,\text {fb}^{-1}$. Jet multiplicity and flavor are used to enhance signal sensitivity, and dedicated control regions are used to constrain the dominant backgrounds. The observed and expected signal significances are, respectively, 1.6 and 1.0 standard deviations, and the $\mathrm {t}\overline{\mathrm {t}} \mathrm {t}\overline{\mathrm {t}}$ cross section is measured to be $16.9^{+13.8}_{-11.4}$ $\,\text {fb}$, in agreement with next-to-leading-order standard model predictions. These results are also used to constrain the Yukawa coupling between the top quark and the Higgs boson to be less than 2.1 times its expected standard model value at 95% confidence level.

## Introduction

**Fig. 1:**
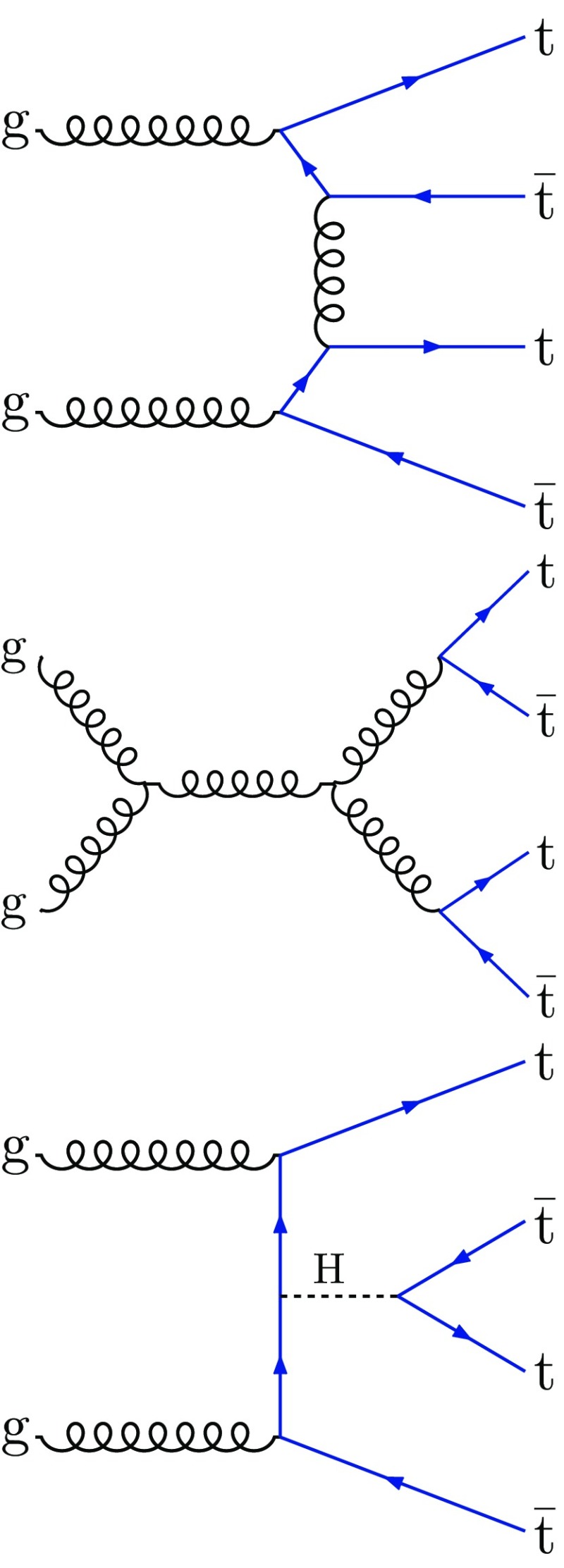
Representative Feynman diagrams for $\mathrm {t}\overline{\mathrm {t}} \mathrm {t}\overline{\mathrm {t}}$ production at LO in the SM

In the standard model (SM) the production of four top quarks ($\mathrm {t}\overline{\mathrm {t}} \mathrm {t}\overline{\mathrm {t}}$) is a rare process, with representative leading-order (LO) Feynman diagrams shown in Fig. 1. Many beyond-the-SM (BSM) theories predict an enhancement of the $\mathrm {t}\overline{\mathrm {t}} \mathrm {t}\overline{\mathrm {t}}$ cross section, $\sigma (\mathrm {p}\mathrm {p}\rightarrow \mathrm {t}\overline{\mathrm {t}} \mathrm {t}\overline{\mathrm {t}}$), such as gluino pair production in the supersymmetry framework [1–10], the pair production of scalar gluons [11, 12], and the production of a heavy pseudoscalar or scalar boson in association with a $\mathrm {t}\overline{\mathrm {t}}$ pair in Type II two-Higgs-doublet models (2HDM) [13–15]. In addition, a top quark Yukawa coupling larger than expected in the SM can lead to a significant increase in $\mathrm {t}\overline{\mathrm {t}} \mathrm {t}\overline{\mathrm {t}}$ production via an off-shell SM Higgs boson [16]. The SM prediction for $\sigma (\mathrm {p}\mathrm {p}\rightarrow \mathrm {t}\overline{\mathrm {t}} \mathrm {t}\overline{\mathrm {t}}$) at $\sqrt{s} = 13 \,\text {TeV}$ is $9.2^{+2.9}_{-2.4}$ $\,\text {fb}$ at next-to-leading order (NLO) [17]. An alternative prediction of $12.2^{+5.0}_{-4.4}$ $\,\text {fb}$ is reported in Ref. [16], obtained from a LO calculation of $9.6^{+3.9}_{-3.5}$ $\,\text {fb}$ and an NLO/LO *K*-factor of 1.27 based on the $14\,\text {TeV}$ calculation of Ref. [18].

After the decays of the top quarks, the final state contains several jets resulting from the hadronization of light quarks and b quarks (b jets), and may contain isolated leptons and missing transverse momentum depending on the decays of the $\mathrm {W}$ bosons [19]. Among these final states, the same-sign dilepton and the three- (or more) lepton final states, considering $\ell = \mathrm {e}, \mu$, correspond to branching fractions in $\mathrm {t}\overline{\mathrm {t}} \mathrm {t}\overline{\mathrm {t}}$ events of 8 and 1%, respectively, excluding the small contribution from $\mathrm {W}\rightarrow \tau \nu$, which is included in selected events. However, due to the low level of backgrounds, these channels are the most sensitive to $\mathrm {t}\overline{\mathrm {t}} \mathrm {t}\overline{\mathrm {t}}$ production in the regime with SM-like kinematic properties. The ATLAS and CMS Collaborations at the CERN LHC have previously searched for SM $\mathrm {t}\overline{\mathrm {t}} \mathrm {t}\overline{\mathrm {t}}$ production in $\sqrt{s}= 8$ and 13$\,\text {TeV}$ $\mathrm {p}$ $\mathrm {p}$ collisions [20–24]. The most sensitive of these results is a re-interpretation of the CMS same-sign dilepton search for BSM physics at 13 $\,\text {TeV}$ [23], with an observed (expected) $\mathrm {t}\overline{\mathrm {t}} \mathrm {t}\overline{\mathrm {t}}$ cross section upper limit (assuming no SM $\mathrm {t}\overline{\mathrm {t}} \mathrm {t}\overline{\mathrm {t}}$ signal) of 42 ($27^{+13}_{-8}$)$\,\text {fb}$ at the 95% confidence level (CL).

The previous search is inclusive, exploring the final state with two same-sign leptons and at least two jets, using an integrated luminosity of 35.9$\,\text {fb}^{-1}$ [23]. The analysis described in this paper is based on the same data set and improves on the previous search by optimizing the signal selection for sensitivity to SM $\mathrm {t}\overline{\mathrm {t}} \mathrm {t}\overline{\mathrm {t}}$ production, by using an improved b jet identification algorithm, and by employing background estimation techniques that are adapted to take into account the higher jet and b jet multiplicity requirements in the signal regions.

## Background and signal simulation

Monte Carlo (MC) simulations at NLO are used to evaluate the $\mathrm {t}\overline{\mathrm {t}} \mathrm {t}\overline{\mathrm {t}}$ signal acceptance and to estimate the background from diboson ($\mathrm {W}$ $\mathrm {Z}$, ZZ, $\mathrm {Z} \mathrm {\gamma }$, $\mathrm {W}^{\pm }\mathrm {W}^{\pm }$) and triboson ($\mathrm {W}$ $\mathrm {W}$ $\mathrm {W}$, $\mathrm {W}$ $\mathrm {W}$ $\mathrm {Z}$, $\mathrm {W}\mathrm {Z} \mathrm {Z}$, $\mathrm {Z} \mathrm {Z} \mathrm {Z}$, $\mathrm {W}\mathrm {W}\mathrm {\gamma }$, $\mathrm {W}\mathrm {Z} \mathrm {\gamma }$) processes, as well as from production of single top quarks ($\mathrm {t} \mathrm {Z} \mathrm {q}$, $\mathrm {t} \mathrm {\gamma }$), or $\mathrm {t}\overline{\mathrm {t}}$ produced in association with a boson ($\mathrm {t}\overline{\mathrm {t}} \mathrm {W}$, $\mathrm {t}\overline{\mathrm {t}} \mathrm {Z}/\gamma ^{*}$, $\mathrm {t}\overline{\mathrm {t}} \text{ H }$). These samples are generated using the NLO MadGraph 5_amc@nlo 2.2.2 [17] program with up to one additional parton in the matrix-element calculation, except for $\mathrm {W}^{\pm }\mathrm {W}^{\pm }$ which is generated with up to two additional partons, and the $\mathrm {W}$ $\mathrm {Z}$, ZZ and $\mathrm {t}\overline{\mathrm {t}} \text{ H }$ samples, which are generated with no additional partons with the powheg box  v2 [25, 26] program. The $\mathrm {t}\overline{\mathrm {t}} \mathrm {Z}/\gamma ^{*}$ sample with $\mathrm {Z}/\gamma ^{*}\rightarrow \ell \ell$ is generated with a dilepton invariant mass greater than $1\,\text {GeV}$. The LO MadGraph 5_amc@nlo generator, scaled to NLO cross sections, is used to estimate the $\mathrm {W}\mathrm {\gamma }$ and $\mathrm {t}\overline{\mathrm {t}} \mathrm {\gamma }$ processes with up to three additional partons. Other rare backgrounds, such as $\mathrm {t}\overline{\mathrm {t}}$ production in association with dibosons ($\mathrm {t}\overline{\mathrm {t}} \mathrm {W}\mathrm {W}$, $\mathrm {t}\overline{\mathrm {t}} \mathrm {W}\mathrm {Z}$, $\mathrm {t}\overline{\mathrm {t}} \mathrm {Z} \mathrm {Z}$, $\mathrm {t}\overline{\mathrm {t}} \mathrm {W}\mathrm {H}$, $\mathrm {t}\overline{\mathrm {t}} \mathrm {Z} \mathrm {W}$, $\mathrm {t}\overline{\mathrm {t}} \mathrm {H} \mathrm {H}$), triple top quark production ($\mathrm {t}\overline{\mathrm {t}} \mathrm {t}$, $\mathrm {t}\overline{\mathrm {t}} \mathrm {t} \mathrm {W}$), and $\mathrm {t} \mathrm {W}\mathrm {Z}$ are generated using LO MadGraph 5_amc@nlo without additional partons, and scaled to NLO cross sections [27]. The NNPDF3.0LO (NNPDF3.0NLO) [28] parton distribution functions (PDFs) are used to generate all LO (NLO) samples. Parton showering and hadronization, as well as $\mathrm {W}^{\pm }\mathrm {W}^{\pm }$ from double-parton-scattering, are modeled by the pythia  8.205 [29] program, while the MLM [30] and FxFx [31] prescriptions are employed in matching additional partons in the matrix-element calculations to parton showers in the LO and NLO samples, respectively. The top quark mass in the generators is set to $172.5\,\text {GeV}$. The Geant4 package [32] is used to model the response of the CMS detector. Additional proton–proton interactions (pileup) within the same or nearby bunch crossings are also included in the simulated events.

To improve the MC modeling of the multiplicity of additional jets from initial-state radiation (ISR), simulated $\mathrm {t}\overline{\mathrm {t}} \mathrm {W}$ and $\mathrm {t}\overline{\mathrm {t}} \mathrm {Z}/\gamma ^{*}$ events are reweighted based on the number of ISR jets ($N_{\text {jets}}^\mathrm {ISR}$). The reweighting is based on a comparison of the light-flavor jet multiplicity in dilepton $\mathrm {t}\overline{\mathrm {t}}$ events in data and simulation. The method requires exactly two jets identified as originating from b quarks in dilepton $\mathrm {t}\overline{\mathrm {t}}$ events, and assumes that all other jets are from ISR. Weighting factors are obtained as a function of $N_{\text {jets}}^\mathrm {ISR}$ to bring data and MC into agreement. These weights are then applied, keeping the total cross section constant, to $\mathrm {t}\overline{\mathrm {t}} \mathrm {W}$ and $\mathrm {t}\overline{\mathrm {t}} \mathrm {Z}/\gamma ^{*}$ MC as a function of the number of jets not originating from top quark, $\mathrm {W}$, or $\mathrm {Z}$ decays. To improve the modeling of the flavor of additional jets, the simulation is also corrected to account for the measured ratio of $\mathrm {t}\overline{\mathrm {t}} \mathrm {b} \overline{\mathrm {b}} / \mathrm {t}\overline{\mathrm {t}} \text {jj}$ cross sections reported in Ref. [33]. More details on these corrections and their uncertainties are provided in Sect. 6.

## The CMS detector and event reconstruction

The central feature of the CMS detector is a superconducting solenoid of 6$\,\text {m}$ internal diameter, providing a magnetic field of 3.8$\,\text {T}$. Within the solenoid volume are a silicon pixel and strip tracker, a lead tungstate crystal electromagnetic calorimeter (ECAL), and a brass and scintillator hadron calorimeter (HCAL), each composed of a barrel and two endcap sections. Forward calorimeters extend the pseudorapidity ($\eta$) coverage provided by the barrel and endcap detectors. Muons are measured in gas-ionization detectors embedded in the steel flux-return yoke outside the solenoid. A more detailed description of the CMS detector, together with a definition of the coordinate system used and the relevant kinematic variables, can be found in Ref. [34].

Events of interest are selected using a two-tiered trigger system [35]. The first level (L1), composed of custom hardware processors, uses information from the calorimeters and muon detectors to select events at a rate of around 100$\,\text {kHz}$ within a time interval of less than 4$\,\mu \text {s}$. The second level, known as the high-level trigger (HLT), consists of a farm of processors running a version of the full event reconstruction software optimized for fast processing, and reduces the event rate to less than 1$\,\text {kHz}$ before data storage.

Events are processed using the particle-flow (PF) algorithm [36], which reconstructs and identifies each individual particle with an optimized combination of information from the various elements of the CMS detector. The energy of photons is directly obtained from the ECAL measurement. The energy of electrons is determined from a combination of the electron momentum at the primary interaction vertex as determined by the tracker, the energy of the corresponding ECAL cluster, and the energy sum of all bremsstrahlung photons spatially compatible with the electron track [37]. The momentum of muons is obtained from the curvature of the corresponding track, combining information from the silicon tracker and the muon system [38]. The energy of charged hadrons is determined from a combination of their momentum measured in the tracker and the matching ECAL and HCAL energy deposits, corrected for the response function of the calorimeters to hadronic showers. The energy of neutral hadrons is obtained from the corresponding corrected ECAL and HCAL energies.

Hadronic jets are clustered from neutral PF candidates and charged PF candidates associated with the primary vertex, using the anti-$k_{\mathrm {T}}$ algorithm [39, 40] with a distance parameter of 0.4. The jet momentum is determined as the vectorial sum of all PF candidate momenta in the jet. An offset correction is applied to jet energies to take into account the contribution from pileup. Jet energy corrections are derived from simulation, and are improved with in situ measurements of the energy balance in dijet, multijet, $\mathrm {\gamma }$+jet and leptonically decaying Z+jet events [41, 42]. Additional selection criteria are applied to each event to remove spurious jet-like features originating from isolated noise patterns in certain HCAL regions. Jets originating from $\mathrm {b}$ quarks are identified as b-tagged jets using a deep neural network algorithm [43], with a working point chosen such that the efficiency to identify a b jet is 55–70% for a jet transverse momentum ($p_{\mathrm {T}}$) between 20 and 400$\,\text {GeV}$. The misidentification rate for a light-flavor jet is 1–2% in the same jet $p_{\mathrm {T}}$ range. The vector ${\vec p}_{\mathrm {T}}^{\text {miss}}$ is defined as the projection on the plane perpendicular to the beams of the negative vector sum of the momenta of all reconstructed PF candidates in an event [44]. Its magnitude, called missing transverse momentum, is referred to as $p_{\mathrm {T}} ^\text {miss}$. The scalar $p_{\mathrm {T}}$ sum of all jets in an event is referred to as $H_{\mathrm {T}}$.

## Event selection and search strategy

The definitions of objects and the baseline event selection follow closely those of Refs. [23, 45]. Electron identification is based on a multivariate discriminant using shower shape and track quality variables, while for muons it is based on the quality of the geometrical matching between the tracker and muon system measurements. Isolation and impact parameter requirements are applied to both lepton flavors, as well as specific selections designed to improve the accuracy of the charge reconstruction. The combined reconstruction and identification efficiency is in the range of 45–70% (70–90%) for electrons (muons), increasing as a function of $p_{\mathrm {T}}$ and converging to the maximum value for $p_{\mathrm {T}}>$ 60$\,\text {GeV}$. The number of leptons ($N_\ell$), the number of jets ($N_\text {jets}$), and the number of b-tagged jets ($N_\text {b}$) are counted after the application of the basic kinematic requirements summarized in Table 1.

**Table 1:** Kinematic requirements for leptons and jets

Object	$p_{\mathrm {T}}$ ($\text {GeV}$ )	$|\eta |$
Electrons	> 20	< 2.5
Muons	> 20	< 2.4
Jets	> 40	< 2.4
b-tagged jets	> 25	< 2.4

Signal events are selected using triggers that require two leptons with $p_{\mathrm {T}} > 8\,\text {GeV}$ and $H_{\mathrm {T}} > 300\,\text {GeV}$. The trigger efficiency is greater than 95% for di-electron ($\mathrm {e}\mathrm {e}$) and electron-muon ($\mathrm {e}\mathrm {\mu }$) events and about 92% for di-muon ($\mathrm {\mu }\mathrm {\mu }$) events. The baseline selections require $H_{\mathrm {T}} > 300\,\text {GeV}$ and $p_{\mathrm {T}} ^\text {miss} >50\,\text {GeV}$, at least two jets ($N_\text {jets} \ge 2$), at least two b-tagged jets ($N_\text {b} \ge 2$), a leading lepton with $p_{\mathrm {T}} > 25\,\text {GeV}$, and a second lepton of the same charge with $p_{\mathrm {T}} > 20\,\text {GeV}$. To reduce the background from Drell–Yan with a charge-misidentified electron, events with same-sign electron pairs with mass below 12$\,\text {GeV}$ are rejected. Events where a third lepton with $p_{\mathrm {T}}$ larger than 5 (7)$\,\text {GeV}$ for muons (electrons) forms an opposite-sign (OS) same-flavor pair with mass below 12$\,\text {GeV}$ or between 76 and 106$\,\text {GeV}$ are also rejected. If the third lepton has $p_{\mathrm {T}} > 20\,\text {GeV}$ and the invariant mass of the pair is between 76 and 106$\,\text {GeV}$, these rejected events are used to populate a $\mathrm {t}\overline{\mathrm {t}} \mathrm {Z}$ background control region (CRZ). The signal acceptance in the baseline region, including the leptonic $\mathrm {W}$ boson branching fraction, is approximately 1.5%. After these requirements, we define eight mutually exclusive signal regions (SRs) and a control region for the $\mathrm {t}\overline{\mathrm {t}} \mathrm {W}$ background (CRW), based on $N_\text {jets}$, $N_\text {b}$, and $N_\ell$, as detailed in Table 2. The observed and predicted yields in the control and signal regions are used to measure $\sigma (\mathrm {p}\mathrm {p}\rightarrow \mathrm {t}\overline{\mathrm {t}} \mathrm {t}\overline{\mathrm {t}}$), following the procedure described in Sect. 7.

**Table 2:** Definitions of the eight SRs and the two control regions for $\mathrm {t}\overline{\mathrm {t}} \mathrm {W}$ (CRW) and $\mathrm {t}\overline{\mathrm {t}} \mathrm {Z}$ (CRZ)

$N_\ell$	$N_\text {b}$	$N_\text {jets}$	Region
2	2	$\le$ 5	CRW
		6	SR1
		7	SR2
		$\ge$ 8	SR3
	3	5, 6	SR4
		$\ge$ 7	SR5
	$\ge$ 4	$\ge$ 5	SR6
$\ge$ 3	2	$\ge$ 5	SR7
	$\ge$ 3	$\ge$ 4	SR8
Inverted Z veto			CRZ

## Backgrounds

The main backgrounds to the $\mathrm {t}\overline{\mathrm {t}} \mathrm {t}\overline{\mathrm {t}}$ process in the same-sign dilepton and three- (or more) lepton final states arise from rare multilepton processes, such as $\mathrm {t}\overline{\mathrm {t}} \mathrm {W}$, $\mathrm {t}\overline{\mathrm {t}} \mathrm {Z}/\gamma ^{*}$, and $\mathrm {t}\overline{\mathrm {t}} \text{ H }$ ($\mathrm {H} \rightarrow \mathrm {WW}$), and single-lepton or OS dilepton processes with an additional “nonprompt lepton”. Nonprompt leptons consist of electrons from conversions of photons in jets and leptons from the decays of heavy- or light-flavor hadrons. In this category we include also hadrons misidentified as leptons. The minor background from OS dilepton events with a charge-misidentified lepton is also taken into account.

Rare multilepton processes are estimated using simulated events. Control regions are used to constrain the normalization of the $\mathrm {t}\overline{\mathrm {t}} \mathrm {W}$ and $\mathrm {t}\overline{\mathrm {t}} \mathrm {Z}$ backgrounds, as described in Sect. 7, while for other processes the normalization is based on the NLO cross sections referenced in Sect. 2. Processes such as the associated production of a $\mathrm {t}\overline{\mathrm {t}}$ pair with a pair of bosons ($\mathrm {W}$, $\mathrm {Z}$, $\mathrm {H}$) are grouped into a “$\mathrm {t}\overline{\mathrm {t}} \mathrm {VV}$” category. Associated photon production processes such as $\mathrm {W}\mathrm {\gamma }$, $\mathrm {Z} \mathrm {\gamma }$, $\mathrm {t}\overline{\mathrm {t}} \mathrm {\gamma }$, and $\mathrm {t} \mathrm {\gamma }$, where an electron is produced in an unidentified photon conversion, are grouped into a “X$\mathrm {\gamma }$” category. All residual processes with very small contributions, including diboson ($\mathrm {W}$ $\mathrm {Z}$, ZZ, $\mathrm {W}^{\pm }\mathrm {W}^{\pm }$ from single- and double-parton scattering), triboson ($\mathrm {W}$ $\mathrm {W}$ $\mathrm {W}$, $\mathrm {W}$ $\mathrm {W}$ $\mathrm {Z}$, $\mathrm {W}\mathrm {Z} \mathrm {Z}$, $\mathrm {Z} \mathrm {Z} \mathrm {Z}$, $\mathrm {W}\mathrm {W}\mathrm {\gamma }$, $\mathrm {W}\mathrm {Z} \mathrm {\gamma }$), and rare single top quark ($\mathrm {t} \mathrm {Z} \mathrm {q}$, $\mathrm {t} \mathrm {W}\mathrm {Z}$) and triple top quark processes ($\mathrm {t}\overline{\mathrm {t}} \mathrm {t}$ and $\mathrm {t}\overline{\mathrm {t}} \mathrm {t} \mathrm {W}$), are grouped into a “Rare” category.

The nonprompt lepton and charge-misidentified lepton backgrounds are estimated following the methods described in Ref. [23]. For nonprompt leptons, an estimate referred to as the “tight-to-loose” method defines two control regions by modifying the lepton identification (including isolation) and event kinematic requirements, respectively. An “application region” is defined for every SR by requiring at least one lepton to fail the standard identification (“tight”) while satisfying a more relaxed one (“loose”). To obtain the nonprompt lepton background estimate in the corresponding SR, the event yield in each application region is weighted by a factor of $\epsilon _\mathrm {TL} / (1-\epsilon _\mathrm {TL})$ for each lepton failing the tight requirement. The $\epsilon _\mathrm {TL}$ parameter is the probability that a nonprompt lepton that satisfies a loose lepton selection also satisfies the tight selection. It is extracted as a function of lepton flavor and kinematic properties from a “measurement region” that consists of a single-lepton events with event kinematic properties designed to suppress the $\mathrm {W}\rightarrow \ell \nu$ contribution.

For charge-misidentified leptons, an OS dilepton control region is defined for each same-sign dilepton signal region. Its yield is then weighted by the charge misidentification probability estimated in simulation, which ranges between $10^{-5}$ and $10^{-3}$ for electrons and is negligible for muons.

## Systematic uncertainties

The sources of experimental and theoretical uncertainty for the data and simulations are summarized in Table 3. The uncertainty in the integrated luminosity is 2.5% [46]. The simulation is reweighted to match the distribution in the number of pileup collisions per event in data. The uncertainty in the inelastic cross section propagated to the final yields provides an uncertainty of at most 6%.

Trigger efficiencies are measured with an uncertainty of 2% in an independent data sample selected using single-lepton triggers. Lepton-efficiency scale factors, used to account for differences in the reconstruction and identification efficiencies between data and simulation, are measured using a “tag-and-probe” method in data enriched in $\mathrm {Z} \rightarrow \ell \ell$ events [37, 38]. The scale factors are applied to all simulated processes with an uncertainty per lepton of approximately 3% for muons and 4% for electrons.

The uncertainty in the calibration of the jet energy scale depends on the $p_{\mathrm {T}}$ and $\eta$ of the jet and results in a 1–15% variation in the event yield in a given SR. The uncertainty due to the jet energy resolution is estimated by broadening the resolution in simulation [42], and the resulting effect is a change of 1–5% in the SR yields. The b tagging efficiency in simulation is corrected using scale factors determined from efficiencies measured in data and simulation [47]. The uncertainty in the measured scale factors results in an overall effect between 1 and 15%, again depending on the SR.

As mentioned in Sect. 2, $\mathrm {t}\overline{\mathrm {t}} \mathrm {W}$ and $\mathrm {t}\overline{\mathrm {t}} \mathrm {Z}/\gamma ^{*}$ simulated events are reweighted to match the number of additional jets observed in data. The reweighting factors vary between 0.92 for $N_{\text {jets}}^\mathrm {ISR}=1$ and 0.77 for $N_{\text {jets}}^\mathrm {ISR} \ge 4$. Half of the difference from unity is taken as a systematic uncertainty in these reweighting factors to cover differences observed between data and simulation when the factors are used to reweight simulation in a control sample enriched in single-lepton $\mathrm {t}\overline{\mathrm {t}}$ events. Uncertainties in the reweighting factors are treated as correlated among regions. Simulated $\mathrm {t}\overline{\mathrm {t}} \mathrm {W}$ and $\mathrm {t}\overline{\mathrm {t}} \mathrm {Z}/\gamma ^{*}$ events with two b quarks not originating from top quark decay are also weighted to account for the CMS measurement of the ratio of cross sections $\sigma ({\mathrm {t}\overline{\mathrm {t}} \mathrm {b} \overline{\mathrm {b}} })/\sigma ({\mathrm {t}\overline{\mathrm {t}} \mathrm {jj}})$, which was found to be a factor of $1.7\pm 0.6$ larger than the MC prediction [33]. In signal regions requiring four b-tagged jets, where the effect is dominant, this results in a systematic uncertainty of up to 15% on the total background prediction. In signal regions requiring three b-tagged jets, the dominant origin of the additional b-tagged jet is a charm quark from a $\mathrm {W}$ decay, so the effect is negligible.

Uncertainties in the renormalization and factorization scales (varied by a factor of two) and from the choice of PDF [48, 49] affect the number of events expected (normalization) in the simulated background processes, as well as the acceptance for the $\mathrm {t}\overline{\mathrm {t}} \mathrm {t}\overline{\mathrm {t}}$ signal. The effects of these uncertainties on the relative distribution of events in the signal regions (shape) are also considered. For the $\mathrm {t}\overline{\mathrm {t}} \mathrm {W}$ and $\mathrm {t}\overline{\mathrm {t}} \mathrm {Z}/\gamma ^{*}$ backgrounds, the normalization uncertainty is 40%, while for $\mathrm {t}\overline{\mathrm {t}} \text{ H }$ a 50% normalization uncertainty reflects the signal strength of $1.5 \pm 0.5$ measured by CMS [50]. The processes in the Rare category along with X$\mathrm {\gamma }$ and $\mathrm {t}\overline{\mathrm {t}} \mathrm {VV}$, many of which have never been observed, are expected to give small contributions to the event yields in the signal regions. We assign separate 50% normalization uncertainties to each of these three categories. The shape uncertainty resulting from variations of the renormalization and factorization scales is as large as 15% for the $\mathrm {t}\overline{\mathrm {t}} \mathrm {W}$, $\mathrm {t}\overline{\mathrm {t}} \mathrm {Z}/\gamma ^{*}$, and $\mathrm {t}\overline{\mathrm {t}} \text{ H }$ backgrounds, and 10% for the $\mathrm {t}\overline{\mathrm {t}} \mathrm {t}\overline{\mathrm {t}}$ signal, while the effect from the PDF is only 1%. For the signal, the uncertainty in the acceptance from variations of the scales (PDFs) is 2% (1%). In addition, for the $\mathrm {t}\overline{\mathrm {t}} \mathrm {t}\overline{\mathrm {t}}$ signal, the scales that determine ISR and final-state radiation (FSR) in the parton shower are also varied, resulting in a 6% change in the acceptance and shape variations as large as 15%.

For nonprompt and charge-misidentified lepton backgrounds, the statistical uncertainty from the application region depends on the SR considered. The background from misidentified charge is assigned a systematic uncertainty of 20%, based on comparisons of the expected number of same-sign events estimated from an OS control sample and the observed same-sign yield in a control sample enriched in $\mathrm {Z} \rightarrow \mathrm {e}^+\mathrm {e}^-$ events with one electron or positron having a misidentified charge.

In addition to the statistical uncertainty, the nonprompt lepton background is assigned an overall normalization uncertainty of 30% to cover variations observed in closure tests performed with simulated multijet and $\mathrm {t}\overline{\mathrm {t}}$ events. This uncertainty is increased to 60% for electrons with $p_{\mathrm {T}} > 50\,\text {GeV}$, to account for trends observed at high $p_{\mathrm {T}}$ in the closure tests. We also include an uncertainty related to the subtraction of events with prompt leptons (from electroweak processes with a $\mathrm {W}$ or $\mathrm {Z}$ boson) in the measurement region, which has an effect between 1% and 50%, depending on the SR. The prompt lepton contamination was also checked in the application region, where it was found to be below 1%.

Experimental uncertainties are treated as correlated among signal regions for all signal and background processes. Systematic uncertainties in data-driven estimates and theoretical uncertainties are treated as uncorrelated between processes, but correlated among signal regions. Statistical uncertainties from the limited number of simulated events or in the number of events in data control regions are considered uncorrelated.

**Table 3:** Summary of the sources of uncertainty and their effect on signal and background yields. The first group lists experimental and theoretical uncertainties in simulated signal and background processes. The second group lists normalization uncertainties in the estimated backgrounds

Source	Uncertainty (%)
Integrated luminosity	2.5
Pileup	0–6
Trigger efficiency	2
Lepton selection	4–10
Jet energy scale	1–15
Jet energy resolution	1–5
$\mathrm {b}$ tagging	1–15
Size of simulated sample	1–10
Scale and PDF variations	10–15
ISR/FSR (signal)	5–15
$\mathrm {t}\overline{\mathrm {t}} \mathrm {H}$ (normalization)	50
Rare, X$\mathrm {\gamma }$, $\mathrm {t}\overline{\mathrm {t}} \mathrm {VV}$ (norm.)	50
$\mathrm {t}\overline{\mathrm {t}} \mathrm {Z}/\gamma ^{*}$, $\mathrm {t}\overline{\mathrm {t}} \mathrm {W}$ (normalization)	40
Charge misidentification	20
Nonprompt leptons	30–60

## Results and interpretation

**Fig. 2:**
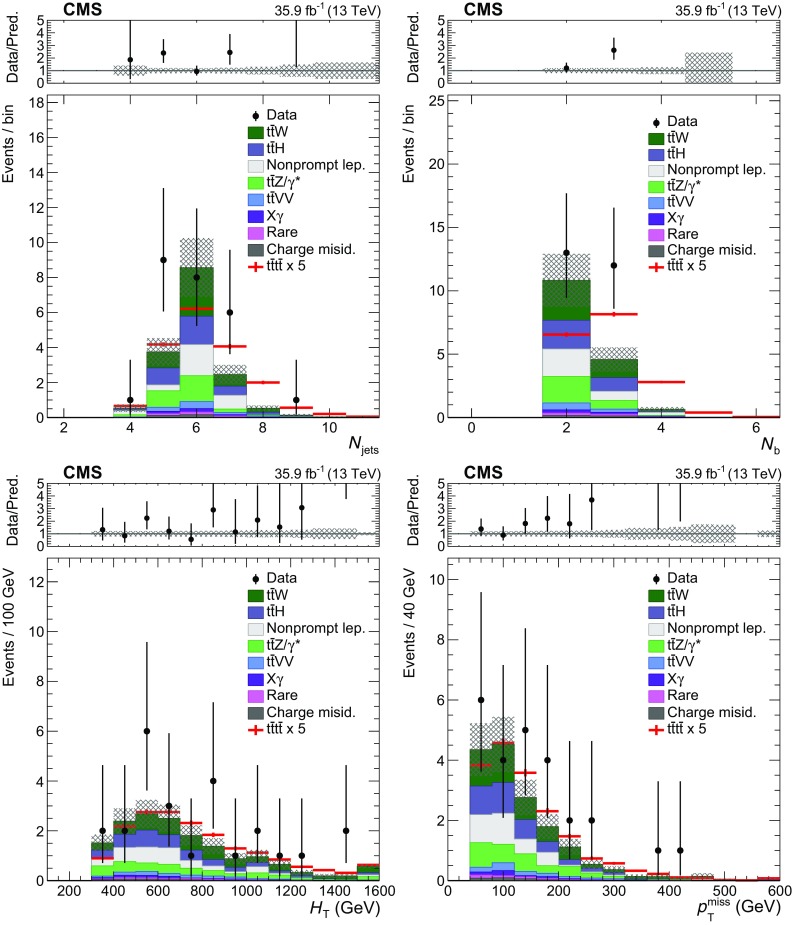
Distributions in $N_\text {jets}$ (upper left), $N_\text {b}$ (upper right), $H_{\mathrm {T}}$ (lower left), and $p_{\mathrm {T}} ^\text {miss}$ (lower right) in the signal regions (SR 1–8), before fitting to data, where the last bins include the overflows. The hatched areas represent the total uncertainties in the SM background predictions, while the solid lines represent the $\mathrm {t}\overline{\mathrm {t}} \mathrm {t}\overline{\mathrm {t}}$ signal, scaled up by a factor of 5, assuming the SM cross section from Ref. [17]. The upper panels show the ratios of the observed event yield to the total background prediction. Bins without a data point have no observed events

**Fig. 3:**
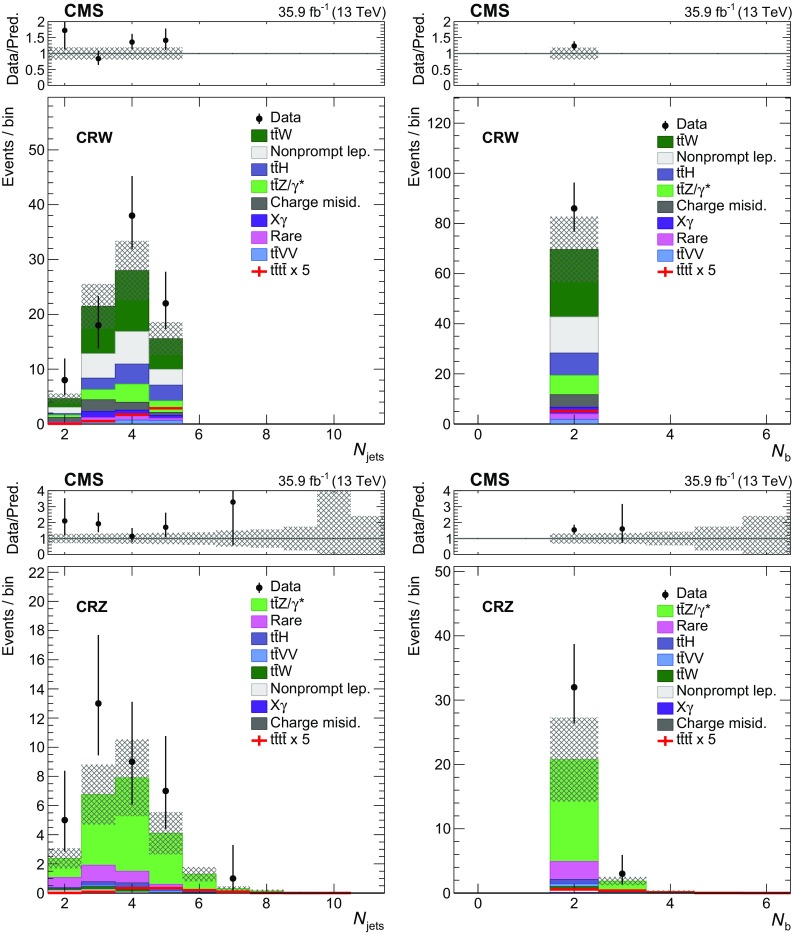
Distributions in $N_\text {jets}$ and $N_\text {b}$ in $\mathrm {t}\overline{\mathrm {t}} \mathrm {W}$ (upper) and $\mathrm {t}\overline{\mathrm {t}} \mathrm {Z}$ (lower) control regions, before fitting to data. The hatched area represents the uncertainty in the SM background prediction, while the solid line represents the $\mathrm {t}\overline{\mathrm {t}} \mathrm {t}\overline{\mathrm {t}}$ signal, scaled up by a factor of 5, assuming the SM cross section from Ref. [17]. The upper panels show the ratios of the observed event yield to the total background prediction. Bins without a data point have no observed events

The properties of events in the signal regions (SR 1–8 as defined in Table 2) are shown in Fig. 2, where distributions of the main kinematic variables in the data ($N_\text {jets}$, $N_\text {b}$, $H_{\mathrm {T}}$, and $p_{\mathrm {T}} ^\text {miss}$) are compared to SM background predictions. The $N_\text {jets}$ and $N_\text {b}$ distributions for CRW and CRZ are shown in Fig. 3. In both figures we overlay the expected SM $\mathrm {t}\overline{\mathrm {t}} \mathrm {t}\overline{\mathrm {t}}$ signal, scaled by a factor of 5. The SM predictions are generally consistent with the observations, with some possible underestimation in CRW and CRZ.

**Fig. 4:**
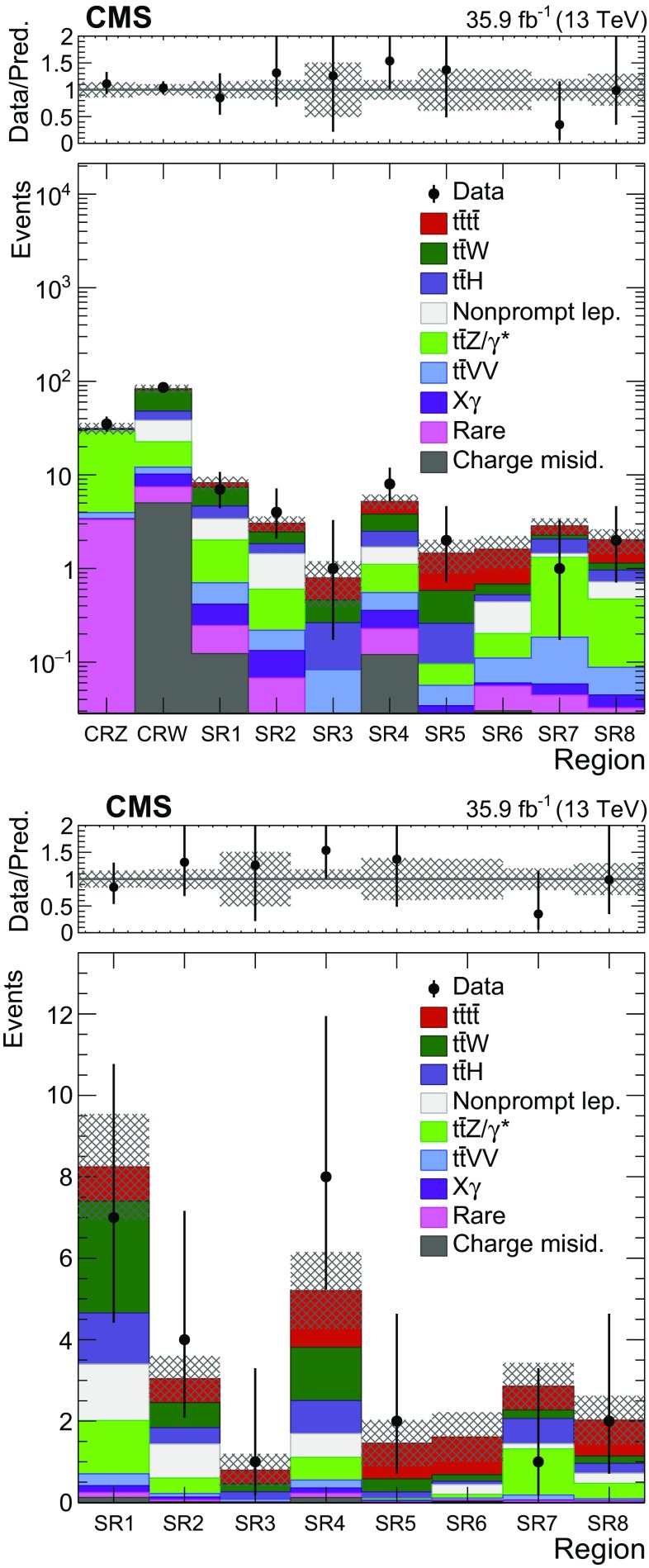
Observed yields in the control and signal regions (upper, in log scale), and signal regions only (lower, in linear scale), compared to the post-fit predictions for signal and background processes. The hatched areas represent the total uncertainties in the signal and background predictions. The upper panels show the ratios of the observed event yield and the total prediction of signal and background. Bins without a data point have no observed events

The yields from SR 1–8, CRW, and CRZ are combined in a maximum-likelihood fit, following the procedures described in Ref. [51], to estimate a best-fit cross section for $\mathrm {t}\overline{\mathrm {t}} \mathrm {t}\overline{\mathrm {t}}$, the significance of the observation relative to the background-only hypothesis, and the upper limit on $\sigma (\mathrm {p}\mathrm {p}\rightarrow \mathrm {t}\overline{\mathrm {t}} \mathrm {t}\overline{\mathrm {t}}$). The experimental and theoretical uncertainties described in Sect. 6 are incorporated in the likelihood as “nuisance” parameters and are profiled in the fit. Nuisance parameters corresponding to systematic uncertainties are parameterized as log-normal distributions. The fitted values of the nuisance parameters are found to be consistent with their initial values within uncertainties. The nuisance parameters corresponding to the $\mathrm {t}\overline{\mathrm {t}} \mathrm {W}$ and $\mathrm {t}\overline{\mathrm {t}} \mathrm {Z}/\gamma ^{*}$ normalizations are scaled by $1.2\pm 0.3$ and $1.3\pm 0.3$, respectively, while other background contributions including $\mathrm {t}\overline{\mathrm {t}} \text{ H }$ are scaled up by 1.1 or less. The signal and control region results after the maximum-likelihood fit (post-fit) are shown in Fig. 4, with the fitted $\mathrm {t}\overline{\mathrm {t}} \mathrm {t}\overline{\mathrm {t}}$ signal contribution added to the background predictions, which are given in Table 4. The $\mathrm {t}\overline{\mathrm {t}} \mathrm {t}\overline{\mathrm {t}}$ cross section is measured to be $16.9^{+13.8}_{-11.4}$ $\,\text {fb}$, where the best-fit value of the parameter and an approximate 68% CL confidence interval are extracted following the procedure described in Sec. 3.2 of Ref. [52]. The observed and expected significances relative to the background-only hypothesis are found to be 1.6 and 1.0 standard deviations, respectively, where the expectation is based on the central value of the NLO SM cross section of $9.2^{+2.9}_{-2.4}$ $\,\text {fb}$ [17]. The observed 95% CL upper limit on the cross section, based on an asymptotic formulation [53] of the modified frequentist $\hbox {CL}_\mathrm {s}$ criterion [54, 55], is found to be 41.7$\,\text {fb}$. The corresponding expected upper limit, assuming no SM $\mathrm {t}\overline{\mathrm {t}} \mathrm {t}\overline{\mathrm {t}}$ contribution to the data, is $20.8^{+11.2}_{-6.9}$ $\,\text {fb}$, showing a significant improvement relative to the value of 27$\,\text {fb}$ of Ref. [23].

**Fig. 5:**
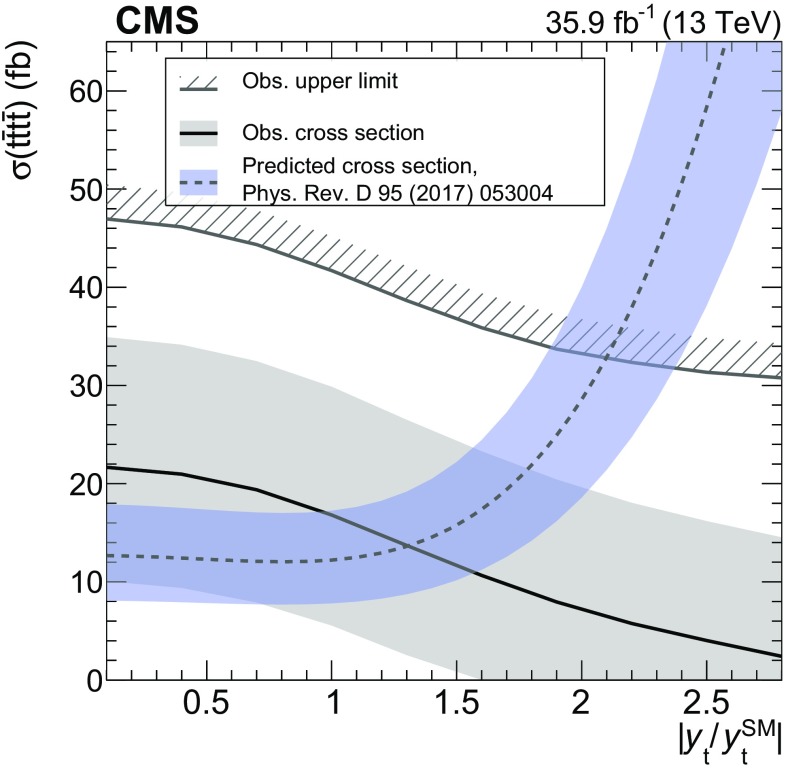
The predicted SM value of $\sigma (\mathrm {p}\mathrm {p}\rightarrow \mathrm {t}\overline{\mathrm {t}} \mathrm {t}\overline{\mathrm {t}}$) [16], calculated at LO with an NLO/LO *K*-factor of 1.27, as a function of $|y_{\mathrm {t}}/y_{\mathrm {t}}^{\mathrm {SM}} |$ (dashed line), compared with the observed value of $\sigma (\mathrm {p}\mathrm {p}\rightarrow \mathrm {t}\overline{\mathrm {t}} \mathrm {t}\overline{\mathrm {t}}$) (solid line), and with the observed 95% CL upper limit (hatched line)

The $\mathrm {p}\mathrm {p}\rightarrow \mathrm {t}\overline{\mathrm {t}} \mathrm {t}\overline{\mathrm {t}}$ process has contributions from diagrams with virtual Higgs bosons, as shown in Fig. 1. Experimental information on $\sigma (\mathrm {p}\mathrm {p}\rightarrow \mathrm {t}\overline{\mathrm {t}} \mathrm {t}\overline{\mathrm {t}}$) can therefore be used to constrain the Yukawa coupling, $y_{\mathrm {t}}$, between the top quark and the Higgs boson. We constrain $y_{\mathrm {t}}$ assuming that the signal acceptance is not affected by the relative contribution of the virtual Higgs boson diagrams. As the cross section for the $\mathrm {t}\overline{\mathrm {t}} \text{ H }$ background also depends on the top quark Yukawa coupling, for the purpose of constraining $y_{\mathrm {t}}$ the fit described above is repeated with the $\mathrm {t}\overline{\mathrm {t}} \text{ H }$ contribution scaled by the square of the absolute value of the ratio of the top quark Yukawa coupling to its SM value ($|y_{\mathrm {t}}/y_{\mathrm {t}}^{\mathrm {SM}} |^2$), where $y_{\mathrm {t}}^{\mathrm {SM}}= m_{\mathrm {t}}( \sqrt{2} G_\mathrm {F})^{1/2} \approx 1$. This results in a dependence of the measured $\sigma (\mathrm {p}\mathrm {p}\rightarrow \mathrm {t}\overline{\mathrm {t}} \mathrm {t}\overline{\mathrm {t}}$) on $|y_{\mathrm {t}}/y_{\mathrm {t}}^{\mathrm {SM}} |$ which is shown in Fig. 5 and is compared to its theoretical prediction. The prediction is obtained from the LO calculation of Ref. [16], with an NLO/LO *K*-factor of 1.27 [18]. The LO calculation is used instead of the NLO one, as Ref. [16] provides a breakdown of the contributions to the cross section according to powers of $y_{\mathrm {t}}$. The prediction also includes the uncertainty associated with varying the renormalization and factorization scales in the LO calculation by a factor of 2. The central, upper and lower values of the theoretical cross section provide respective 95% CL limits for $|y_{\mathrm {t}}/y_{\mathrm {t}}^{\mathrm {SM}} | < 2.1$, <1.9 and <2.4.

**Table 4:** The post-fit background, signal, and total yields with their total uncertainties and the observed number of events in the control and signal regions in data

	SM background	$\mathrm {t}\overline{\mathrm {t}} \mathrm {t}\overline{\mathrm {t}}$	Total	Observed
CRZ	31.7 ± 4.6	0.4 ± 0.3	32.1 ± 4.6	35
CRW	83.7 ± 8.8	1.9 ± 1.2	85.6 ± 8.6	86
SR1	7.7 ± 1.2	0.9 ± 0.6	8.6 ± 1.2	7
SR2	2.6 ± 0.5	0.6 ± 0.4	3.2 ± 0.6	4
SR3	0.5 ± 0.3	0.4 ± 0.2	0.8 ± 0.4	1
SR4	4.0 ± 0.7	1.4 ± 0.9	5.4 ± 0.9	8
SR5	0.7 ± 0.2	0.9 ± 0.6	1.6 ± 0.6	2
SR6	0.7 ± 0.2	1.0 ± 0.6	1.7 ± 0.6	0
SR7	2.3 ± 0.5	0.6 ± 0.4	2.9 ± 0.6	1
SR8	1.2 ± 0.3	0.9 ± 0.6	2.1 ± 0.6	2

## Summary

The results of a search for standard model (SM) production of $\mathrm {t}\overline{\mathrm {t}} \mathrm {t}\overline{\mathrm {t}}$ at the LHC have been presented, using data from $\sqrt{s} = 13\,\text {TeV}$ proton–proton collisions corresponding to an integrated luminosity of 35.9$\,\text {fb}^{-1}$, collected with the CMS detector in 2016. The analysis strategy uses same-sign dilepton as well as three- (or more) lepton events, relying on jet multiplicity and jet flavor to define search regions that are used to probe the $\mathrm {t}\overline{\mathrm {t}} \mathrm {t}\overline{\mathrm {t}}$ process. Combining these regions yields a significance of 1.6 standard deviations relative to the background-only hypothesis, and a measured value for the $\mathrm {t}\overline{\mathrm {t}} \mathrm {t}\overline{\mathrm {t}}$ cross section of $16.9^{+13.8}_{-11.4}$ $\,\text {fb}$, in agreement with the standard model predictions. The results are also re-interpreted to constrain the ratio of the top quark Yukawa coupling to its SM value, yielding $|y_{\mathrm {t}}/y_{\mathrm {t}}^{\mathrm {SM}} | < 2.1$ at 95% confidence level.
